# A Systematic Review and Meta-Analysis Investigating the Relationship between Exposures to Chemical and Non-Chemical Stressors during Prenatal Development and Childhood Externalizing Behaviors

**DOI:** 10.3390/ijerph17072361

**Published:** 2020-03-31

**Authors:** Frances M. Nilsen, Jessica Frank, Nicolle S. Tulve

**Affiliations:** 1 Oak Ridge Institute for Science and Education, U.S. Environmental Protection Agency, Office of Research and Development, 109 TW Alexander Dr., Research Triangle Park, NC 27709, USA; Jessica.Frank1@tetratech.com; 2 U.S. Environmental Protection Agency, Office of Research and Development, 109 TW Alexander Dr., Research Triangle Park, NC 27709, USA; tulve.nicolle@epa.gov

**Keywords:** children, gestational, mental health, cortisol, psychosocial, well-being

## Abstract

Childhood behavioral outcomes have been linked to low quality intrauterine environments caused by prenatal exposures to both chemical and non-chemical stressors. The effect(s) from the many stressors a child can be prenatally exposed to may be influenced by complex interactive relationships that are just beginning to be understood. Chemical stressors influence behavioral outcomes by affecting the monoamine oxidase A (MAOA) enzyme, which is involved in serotonin metabolism and the neuroendocrine response to stress. Non-chemical stressors, particularly those associated with violence, have been shown to influence and exacerbate the externalizing behavioral outcomes associated with low MAOA activity and slowed serotonin metabolism. The adverse developmental effects associated with high stress and maternal drug use during pregnancy are well documented. However, research examining the combined effects of other non-chemical and chemical stressors on development and childhood outcomes as a result of gestational exposures is scarce but is an expanding field. In this systematic review, we examined the extant literature to explore the interrelationships between exposures to chemical and non-chemical stressors (specifically stressful/traumatic experiences), MAOA characteristics, and childhood externalizing behaviors. We observed that exposures to chemical stressors (recreational drugs and environmental chemicals) are significantly related to externalizing behavioral outcomes in children. We also observed that existing literature examining the interactions between MAOA characteristics, exposures to chemical stressors, and traumatic experiences and their effects on behavioral outcomes is sparse. We propose that maternal stress and cortisol fluctuations during pregnancy may be an avenue to link these concepts. We recommend that future studies investigating childhood behaviors include chemical and non-chemical stressors as well as children’s inherent genetic characteristics to gain a holistic understanding of the relationship between prenatal exposures and childhood behavioral outcomes.

## 1. Introduction

The developmental origins of childhood mental and behavioral health outcomes have been an area of increasing research interest over the last 20 years [1,2,3,4]. Childhood outcomes research evolved from the developmental origins of adult disease (DOHaD) hypothesis, which states that numerous adult diseases can be programmed in utero [5,6]. Many adverse childhood outcomes have been attributed to low birth weight, which is considered an indicator of a low-quality intrauterine environment commonly associated with maternal adversity, stress, and drug use [7,8]. Maternal stress during pregnancy and increased maternal stress hormones (i.e., cortisol) have been linked to fetal morphological changes, pregnancy complications, and decreased infant cognitive development [9,10,11]. Maternal drug use during pregnancy is often associated with altered neurodevelopment and behavioral outcomes [8,9,12]. 

Apart from maternal factors, prenatal chemical exposures from both human epidemiology and lab-based animal studies consistently report a relationship to adverse mental health outcomes, including externalizing behaviors such as attention deficit hyperactivity disorder (ADHD), conduct disorders (CD), oppositional defiant disorder (ODD), and aggressive behavior [13,14,15,16,17,18,19,20,21,22,23]. The advanced function and biomolecular composition of the human brain make it particularly susceptible to toxic chemicals and associated neuropsychiatric diseases [24]. Chemical exposures can influence externalizing behavioral outcomes through competitive inhibition of the monoamine oxidase A (MAOA) enzyme involved in metabolism of biogenic amine neurotransmitters and the neuroendocrine response to stress [25,26,27,28,29,30]. The idea that the MAOA gene may influence vulnerability to chemical stressors has been summarized extensively in the literature [31,32,33]. 

Non-chemical stressors, particularly those associated with violence, have been shown to influence and exacerbate childhood externalizing behavioral outcomes associated with MAOA [1,32,34,35,36,37]. A large body of work has examined the interactions between childhood traumatic/stressful experiences and MAOA characteristics with regard to externalizing outcomes, but little research has been done prior to an externalizing diagnosis or in tandem with exposures to chemical and non-chemical stressors [32,34,35,36,38,39,40,41,42,43]. Analyses combining MAOA characteristics with chemical and non-chemical stressors could elucidate additional details that influence behavioral outcomes linked to both MAOA characteristics and stressor exposures.

A variety of studies have examined the pathology and plasticity associated with the quality of the developmental environment, and observed that there are ranges of biochemical, nutritional, and hormonal fluctuations that result in the same developmental outcome [2]. The potential additive effect from prenatal exposures to multiple stressors is subject to the complex interactions described by the conceptual child health and well-being framework put forth by Tulve [44]. The conceptual framework highlights that exposures to chemical and non-chemical stressors from the built, natural, and social environments, combined with inherent characteristics such as genetic predisposition, can affect a child throughout their life course. 

The complex interactions that may arise from prenatal exposures could be mediated via maternal cortisol concentration. Cortisol is the main stress hormone that interacts with the brain to control mood, regulate blood pressure, and manage the use of carbohydrates, fats, and proteins [45]. The cortisol response to diurnal cycles is mediated by the hypothalamus-pituitary-adrenal (HPA) axis. Psychosocial stress is a potent activator of the HPA axis, which elicits the cortisol response to various stressors in the everyday human environment [46]. Cortisol measurements have been investigated in relation to traumatic events and traumatic stress disorders in a variety of cohorts [47,48]. The perceived danger, or anticipatory cognitive appraisal, of stressful events has been shown to explain 35% of the variance in cortisol concentration associated with the stress response [46]. During pregnancy, maternal cortisol concentrations increase progressively through the end of gestation [49]. The amount of perceived stress during pregnancy is associated with maternal cortisol concentrations and more stressful events elicit higher cortisol concentration [49,50,51].

This review builds on the framework put forth by Tulve [44] by examining the current epidemiological literature for interrelationships that may exist between prenatal exposures to chemical and non-chemical stressors as well as the mediating effects of genetic variation and maternal health status on the development of mental health outcomes in children. Furthermore, the results identify existing research gaps and highlight the need for interdisciplinary research to better understand how chemical and non-chemical stressors interact with genetics to affect children’s behavioral health outcomes.

## 2. Materials and Methods 

This review followed the Preferred Reporting Items for Systematic Reviews and Meta-Analyses (PRISMA) guidelines [52,53]. Included references were peer-reviewed, written in English, published between 1980 and 2017, classified as research on children from preschool age (2−5 years) through adolescence (13−18 years) as defined by the PubMed Medical Subject Headings, and used the Diagnostic and Statistical Manual of Mental Disorders (DSM) mental health classification system or a comparable system [54]. In 1980, the American Psychological Association (APA) changed the name and definition of hyperkinetic impulse disorder to attention deficit disorder (ADD) with subtypes relating to hyperactivity, and later to ADHD with no subtypes. Since ADHD is a well-known externalizing behavior, and 1980 marks the beginning of the diagnosis of ADHD as we understand it today, we used 1980 as the starting point for this review [55,56]. 

### 2.1. Search Details 

**Figure 1:**
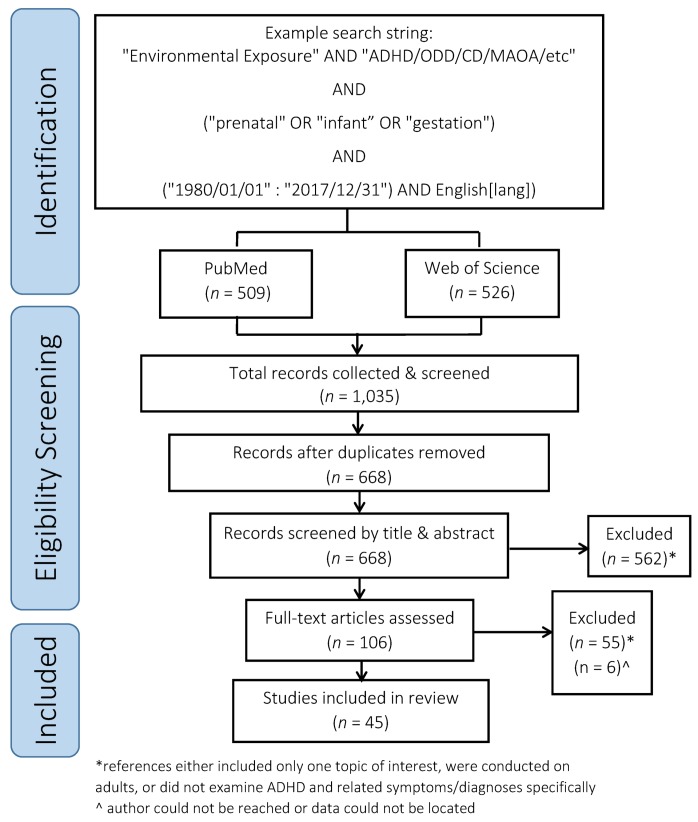
PRISMA diagram of the reference selection process for this review.

Web of Science (Core Collection) and PubMed were the two databases used for identification of relevant primary references using search strings related to “environmental exposure” AND “disruptive behavior disorders” AND “monoamine oxidase.” An example of the search and subsequent screening process can be found in Figure 1 and is detailed in Appendix A. All references included in the meta-analysis were located using this search string. 

### 2.2. Reference Screening Process

The initial screening included review of the title and abstract of each reference. Inclusion criteria for the title and abstract screening were determined based on responses to the following three questions.

1. Does the study include MAOA, childhood externalizing behaviors, and prenatal exposures to chemical and/or non-chemical stressors?
2. Does the paper report a relationship between the topics?
3. Was an externalizing behavioral health outcome the focus of this study?

References meeting all criteria questions were included in the full text screening. The full text screening excluded references that did not focus on an interactive relationship. Studies focused on other mental health outcomes, terrorist events, physical illnesses, or indirect relationships were excluded. Included references that did not provide enough information related to the original study design and/or statistical analysis were marked, and authors were contacted to obtain the necessary information for inclusion in our meta-analysis. If the authors could not be reached, the study was removed from the review (Figure 1).

### 2.3. Data Extraction and Analysis

The prenatal data included in this meta-analysis resulted from the search criteria to examine MAOA characteristics in relation to stressors and behavioral health outcomes. There was a paucity of prenatal studies where MAOA was examined, particularly when compared to the data examined in our childhood exposure publication [30]. As such, the few prenatal MAOA studies included in this review are discussed in detail below. 

Chemical exposure data (including any details regarding blood/urine measurements or survey data), the effect size, standard error, variance, and confidence interval were extracted from relevant tables, graphs, and text within each reference. The extracted data were put into Microsoft Excel (Microsoft Office 2018) and sorted according to exposure and outcome details. All effect size data were converted to odds ratios (OR) for meta-analysis and visualization using R Studio version 3.6.1 and the analysis packages “*metafor*”, “*metaviz*”, and “*ggplot2*” [57,58,59,60,61].

### 2.4. Quality Assessment

The screening process described in Section 2.2 was conducted on 1035 references initially identified on two separate occasions by multiple researchers. Any discrepancies between the two separate screenings were reconciled based on the relevance of the reference’s content to this review. The extracted data mentioned in Section 2.3 were checked for accuracy by two individual reviewers on separate occasions. 

#### 2.4.1. Precision of Evidence

The precision of the individual datasets included in this review was determined using the Grades of Recommendation, Assessment, Development, and Evaluation (GRADE) approach [62]. The GRADE method determines a quality ranking and is based on the imprecision, indirectness, and risk of bias of the data presented in the reference [62,63]. The summary of findings table was created using the freely available Guideline Development Tool (GDT) software [64]. The individual rankings were used to determine the overall risk of imprecision in the meta-analysis, which was a contributing factor in the certainty of evidence presented in the summary of findings table. The individual GRADE quality rankings reported were based on relevance to this review and were not an evaluation of the scientific merit of the included peer-reviewed references, so the individual rankings are not reported. The summary of findings table presents the anticipated absolute effects of the risk of onset of the externalizing behavior compared to the incidence in the U.S. population with a similar diagnosis. The risk related to chemical exposure is based on the pooled odds ratio (OR) from the included studies. 

#### 2.4.2. Bias

The risk of publication bias in the meta-analyses was determined by assessing the symmetry of the data by using the inverse number of participants in each original study (1/*n*) and the standard error (SE) for comparison to the log odds ratio (OR). The statistical presence of asymmetry indicates that there is publication bias present, as non-biased data would have an equal distribution of results on both sides of the line of no effect (1.0) [65,66,67]. Due to the correlation between the SE and the magnitude of the OR (which can lead to inflated type 1 error), we used the 1/*n* method of bias assessment. The 1/*n* method removes the likelihood of type 1 error being an artifact of the SE and OR correlation, and decreases the likelihood of asymmetry due to between study heterogeneity and/or small study effects [66]. Both metrics are presented for the data in this review, but we use the 1/*n* method to determine if publication bias is present in our analyses based on the strengths of that method.

Two independent reviewers assessed the risk of bias across this entire review with the Risk of Bias in Systematic Reviews (ROBIS) tool for etiology-based systematic reviews [68]. The combined assessment indicated that there was a low risk of bias in this meta-analysis. 

### 2.5. Statistical Analysis

#### 2.5.1. Data Grouping

Chemical stressors were separated into two groups: recreational drugs (cigarette smoke, cocaine, alcohol, marijuana, methamphetamine) and environmental chemicals [phthalates/plasticizers (PhPl), organic contaminants (OC), metals (mercury (Hg), lead (Pb))], and further divided into subgroups based on externalizing behavior outcomes if three or more references examined the same diagnosis. The PhPl and OC studies were separated because they included large amounts of data and because the PhPl literature examined and reported sex-specific effects, whereas the OC literature did not. Since these chemicals act on the endocrine system, sex-specific data were analyzed separately when available [69]. The OR data extracted from each reference was used in the relevant meta-analysis, and the general estimate of externalizing behaviors in relation to prenatal exposures to chemicals is reported in the meta-analysis OR statistics in this review. 

#### 2.5.2. Meta-Analysis

Random effects meta-analyses (RMA) were conducted using R Studio and the “*metafor*” package for each class of chemicals. The R Studio syntax using the “*rma*” function is presented in Appendix A. The RMA model was chosen for analysis because it accounts for random error and between study variation [70]. The RMA model was calculated using the restricted (residual) maximum likelihood (REML) estimator, which provides a greater estimate of variance than other estimators [71]. The heterogeneity measures used in this review are *T*^2^ and *I*^2^, which provide a value to accompany the certainty of meta-analytic results. *T*^2^ provides the variance of studies within the meta-analysis; *I*^2^ can be used to compare the current meta-analyses to other relevant meta-analyses [72]. Greater detail regarding the use of *T*^2^ and *I*^2^ can be found in Nilsen and Tulve [30].

The *I*^2^ heterogeneity percentage can be interpreted as:

0% to 40%: heterogeneity might not be important
30% to 60%: may represent moderate heterogeneity
50% to 90%: may represent substantial heterogeneity
75% to 100%: considerable heterogeneity [73].

The meta-analytic results are presented with forest plots that were created using R Studio and the “*metaviz*” package (SI). In a forest plot, the included references are listed on the Y-axis and the corresponding OR is provided on the X-axis. The blue boxes corresponding to each reference’s OR are sized based on the weight of each study/sampling event (based on statistical significance, variance, and number of participants) [72]. The horizontal error bars represent the upper and lower 90% confidence intervals (90% _CI_) that were calculated from the original studies to reflect the variance in the dataset (as most studies presented the 95%_CI_). The meta-analysis summary OR is provided by a red diamond and a vertical dashed red line. The ‘line of no effect’ (the line at which the odds of an outcome occurring are equal to the odds of the outcome not occurring) is represented by a dotted vertical line at an OR of 1.

#### 2.5.3. Meta-Regression

When the *I*^2^ value was above 75% (indicating ‘considerable’ heterogeneity) in the meta-analyses, a meta-regression was performed to investigate the sources of residual heterogeneity. Meta-regression calculations were performed using R Studio and the “*metafor*” package using the “*rma*” function with *“mods=”* added for variable delineation (Appendix A). All variables represented across all relevant data were examined. The meta-regression *I*^2^ values (*I*^2^*_Reg_*) were compared to the *I*^2^ values from the meta-analysis (*I*^2^*_RMA_*) to determine if any of the heterogeneity was attributed to the examined variables. Meta-regression bubble plots were created using R Studio with the *“ggplot2”* package and are presented in the SI when appropriate. 

#### 2.5.4. Sensitivity Analysis

The robustness of data was examined post-hoc using seven meta-analytic estimation methods [74]. The seven estimation methods were the REML, the maximum likelihood (ML), the fixed-effects (FE), the Hedges (HE), the DerSimonian–Laird (DL), the empirical Bayesian (EB), and the Paul–Mandel (PM) estimators. Data robustness is expressed as a percentage, which reflects the number of estimators that yielded ORs within 15% of each other (Table S1). All combined externalizing behavioral health outcome results presented in this review were robust in the sensitivity analysis, with at least 70% agreement between estimation models, except the PhPl data (57%) (Table S1). The diagnosis specific behavioral health outcome results in this review were robust in the sensitivity analysis, with at least 85% agreement between the estimation models, except the ADHD-specific cigarette smoke exposure data (43%) and the male PhPl exposure data (43%) likely due to the variation in the data of those categories (Table S1).

## 3. Results

Forty-five references examining externalizing behaviors related to prenatal exposures to chemical and non-chemical stressors and/or MAOA genotype were included in this systematic review and meta-analysis. Thirty-one references examined prenatal exposures to recreational drugs (21 = cigarette smoke; 6 = alcohol; 3 = cocaine; 1 = marijuana; 1 = methamphetamine; 1 = antidepressants) and 17 examined prenatal exposures to environmental chemicals (9 = OC; 4 = PhPl; 3 = Hg; 1 = Pb). Only the references examining PhPl exposures provided sex-specific data. Two references examined the relationship between MAOA and exposures. One reference examined the relationship between prenatal exposures to chemical stressors, MAOA, and externalizing behaviors in childhood. There were six references that examined prenatal exposures to chemical stressors in relation to traumatic experiences. Individual categories totaled more than 45 as some references included more than one exposure. All chemical stressor data were statistically analyzed except for marijuana, methamphetamine, pharmaceuticals, and Pb exposures because there were less than three references in each of those subgroups. Due to the range of traumatic experience data and the limited MAOA data, a statistical analysis was not conducted on those categories; instead, these data were qualitatively assessed.

### 3.1. Summary of Exposures to Chemical Stressors and Externalizing Behaviors Findings

When the chemical stressor data were separated by exposure type, the cumulative OR from the RMA meta-analyses highlight that prenatal exposures to chemical stressors were consistently associated with greater incidences of externalizing behaviors in children (Table 1).

The publication bias in each meta-analysis was assessed using the 1/*n* method described in Section 2.4. Using this method, the publication bias was statistically significant for the combined externalizing behavioral health outcomes data related to cocaine, alcohol, and organic contaminant exposures (Table S1). Since these datasets were robust above 70% in the sensitivity analysis, their significant publication bias suggests that negative results are not as prevalent in the literature as the positive associations we observed. The male specific PhPl exposures data was also subject to publication bias, but since that dataset was not as robust as the others, we speculate that there may be a data gap at the nexus of prenatal male PhPl exposure, MAOA characteristics and childhood externalizing behavioral health outcomes. 

Using the SE method, all environmental chemical exposure meta-analyses were subject to publication bias, but not the recreational drug exposure meta-analyses (Table S1). Even though there is greater likelihood of type 1 error, and small study effects in the SE method, the dichotomous result is interesting as there is less contaminant exposure data available compared to recreational drug exposure data. However, the differing results between the two methods indicate there is likely some publication bias in each meta-analysis. Unfortunately, publication bias is a caveat of meta-analytic research, as negative data are often difficult to publish, or are not published at all, and the ‘grey’ literature (e.g., conference abstracts, non-peer reviewed sources) seldom presents comprehensive results that can be used for additional analyses.

The precision of the included data was evaluated based on the imprecision of the original studies, specifically the width of reported confidence intervals, as well as the original number of participants being <1000 in the original studies (Table 1, Table S2, Table S3, Table S4). For our meta-analysis, the recreational drug exposure data was determined to be of “moderate” precision; the prenatal PhPl exposure data was determined to be of “moderate” precision when the sexes were separated and “low” precision when the sexes were combined; and, the environmental chemical exposure data differed based on the specific chemical examined (Table 1, Table S5). The certainty of the prenatal exposures to OCs data was “low” due to the imprecision of the original data (Table 1, Table S6). The prenatal Hg data was determined to be “moderate” (Table 1, Table S7).

### 3.2. Prenatal Exposures to Recreational Drugs

#### 3.2.1. Cigarette Smoke

The cigarette smoke data were composed of 21 studies that included a total of 58 sampling events with measurements of prenatal exposures to smoke determined by maternal survey data [16,75,76,77,78,79,80,81,82,83,84,85,86,87,88,89,90,91,92,93,94]. Four of these studies included exposure data that were specific to the first trimester and included 14 sampling events [80,84,88,94]. The data specific to the first trimester are of interest as this period of gestation is the most sensitive to stressors [95,96].

**Figure 2:**
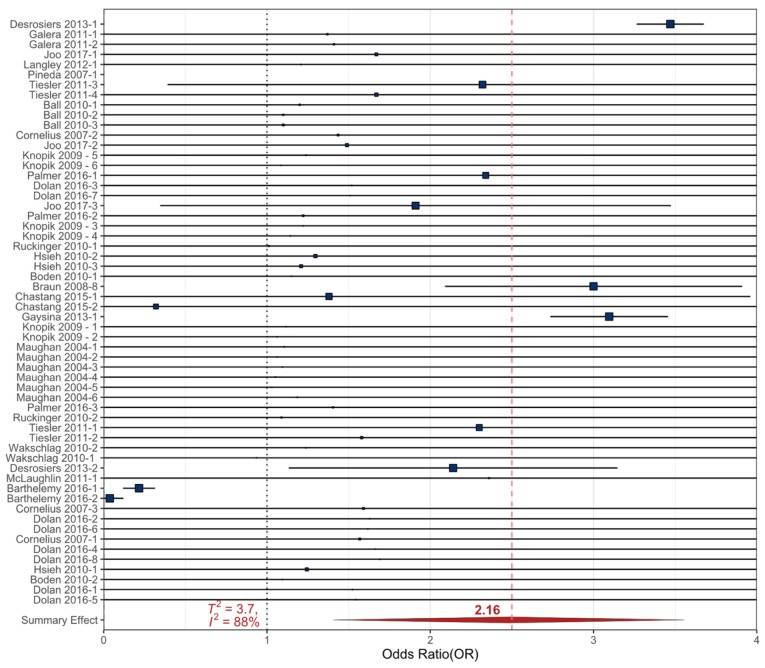
Forest plot showing the random effects meta-analysis for prenatal exposure to cigarette smoke and all childhood externalizing behavioral health outcomes. The squares represent the mean OR from each sampling event (Table S2), horizontal bars are the upper and lower 90% CI, the size variation of the boxes represents the weight each dataset had in the meta-analysis with a larger size indicating larger weights. The diamond and dashed line represent the summary effect OR from the meta-analysis. The ‘line of no effect’ at an OR of 1 is indicated with a dotted line.

The relationship between externalizing behavioral health outcomes and general prenatal cigarette smoke exposures, as well as first trimester exposures, were examined in participants aged 3–18 years using the RMA meta-analysis. The summary effect OR for general prenatal exposures was statistically significant at 2.16 (90%_CI_ 1.36–2.96, *p* < 0.0001), with considerable heterogeneity (*T*^2^ = 3.7, *I*^2^ = 88%) (Table 1, Figure 2). The smaller, first trimester exposure data were related to externalizing behavioral health outcomes with a summary effect OR of 1.45 (90%_CI_ 0.23–2.66) and no heterogeneity (*T*^2^ = 0, *I*^2^ = 0%) or statistical significance due to the variation between the included datasets.

The considerable heterogeneity in the general prenatal exposure meta-analysis provided the opportunity to conduct a meta-regression to determine the influence of the variables from the original studies. The variables that were analyzed in the meta-regression were behavioral health outcomes and number of participants (*n*) in the original study (Figure S1). The variables that significantly influenced the meta-regression were the behavioral health outcomes ADHD (*p* < 0.0001) and general externalizing behaviors (*p* = 0.04). The number of participants was not statistically significant. The identification of the influence of ADHD and externalizing behaviors did reduce the heterogeneity from *I^2^_RMA_* = 88% to *I^2^_Reg_* = 74% and from *T^2^_RMA_ = 3.7 to T^2^_Reg_ =* 3.1. The meta-regression model did account for 17% of the heterogeneity in the data (Figure S1).

Since the meta-regression was not able to identify a specific externalizing behavioral health outcome causing the heterogeneity, the exposure data that reported either ADHD or CD/ODD as an outcome were separated and re-analyzed by meta-analysis as each included 24 sampling events from 13 studies. The externalizing outcomes were not re-analyzed because there were less than three original studies that identified it as an outcome. The follow-up meta-analysis examining the relationship between prenatal cigarette smoke exposures and CD/ODD had a statistically significant summary effect OR of 2.61 (90%_CI_ 2.08–3.14, *p* < 0.001) with negligible heterogeneity (*T*^2^ = 0, *I*^2^ = 0.3%). The follow-up meta-analysis examining the relationship between prenatal cigarette smoke exposures and ADHD outcomes had a statistically significant summary effect OR of 2.76 (90%_CI_ 1.44–4.07, *p* < 0.001), but the heterogeneity in the data was not resolved (*T*^2^ = 5.4, *I*^2^ = 84%). In a final effort to clarify the prenatal cigarette smoke exposure data, a meta-regression was conducted on the 24 sampling events specific to ADHD outcomes. In the ADHD meta-regression, inattention, hyperactivity, and complete ADHD diagnoses were separated. Only the complete ADHD diagnoses were statistically significant and accounted for 9% of the heterogeneity in the data, but the overall heterogeneity was only slightly reduced and still considerable (*T*^2^ = 4.9, *I*^2^ = 80%). Despite the variation in the original data, the statistically significant cumulative ORs and CIs suggest that prenatal cigarette smoke exposure may lead to an increase in the incidence of ADHD, CD/ODD, and other externalizing behavioral health outcomes in children (Table 1). 

#### 3.2.2. Cocaine

**Figure 3:**
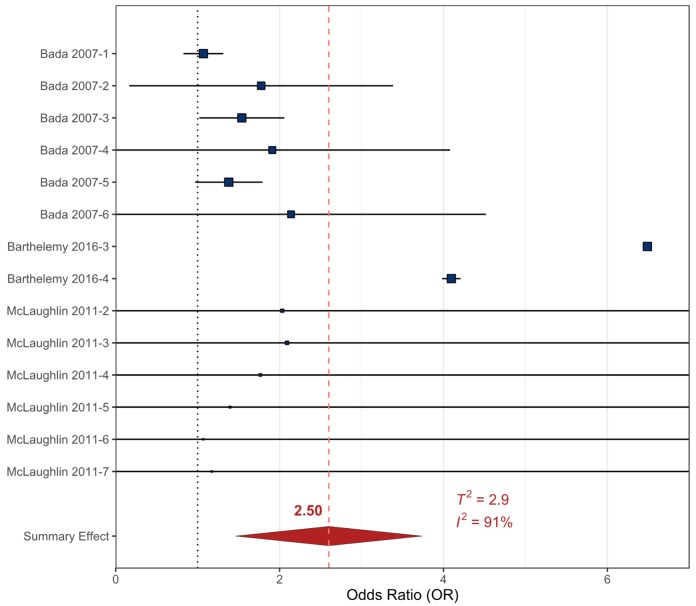
Forest plot showing the externalizing behavioral health outcomes included in the random effects meta-analysis for prenatal exposure to cocaine. The squares represent the mean OR from each reference dataset (Table S3), horizontal error bars represent the upper and lower 90% CI, the size variation of the boxes represents the weight each dataset had in the meta-analysis with a larger size indicating larger weights. The diamond and dashed line represent the summary effect OR based on the meta-analysis. The ‘line of no effect’ at an OR of 1 is indicated with a dotted line.

The prenatal cocaine data were composed of three studies that included a total of 14 sampling events. The measurements of prenatal cocaine exposure were determined by a combination of measurements of cocaine metabolites in maternal/infant urine/infant meconium and/or maternal survey data [76,87,97]. The included studies examined different behavioral outcomes classified as externalizing disorders including aggression, ADHD, CD, ODD, and collective externalizing behaviors in participants aged 3−9 years (Table S3). All externalizing behavioral health outcomes related to prenatal cocaine exposure were analyzed together in the RMA meta-analysis. The summary effect OR for prenatal cocaine exposures on the development of externalizing behavioral outcomes in childhood was statistically significant at 2.50 (90%_CI_ 1.52–3.48, *p* < 0.001), but there was considerable heterogeneity present in the data (*T^2^* = 2.9, *I^2^* = 91%) (Table 1, Figure 3). 

A meta-regression was conducted to further examine the potential sources of heterogeneity. The number of participants sampled in the original study was the only variable present across all datasets and was observed to be statistically significant in the meta-regression model (*p* < 0.0001). This finding indicates that the number of participants can influence the OR in studies such as those included in this model and this variable accounted for 67% of the reduced heterogeneity (*T*^2^*_Reg_* = 1.0, *I*^2^*_Reg_* = 74%. *T*^2^*_RMA_* = 2.9, *I*^2^*_RMA_* = 91%). However, the OR suggests that prenatal cocaine exposure may lead to a slight increase in childhood externalizing behaviors (Table 1). 

#### 3.2.3. Alcohol

**Figure 4:**
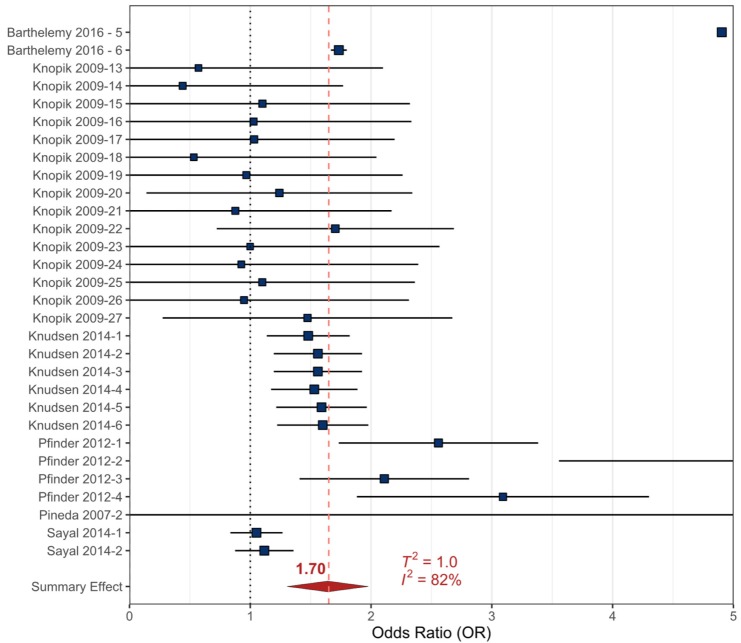
Forest plot showing the externalizing behavioral health outcomes included in the random effects meta-analysis for prenatal exposure to alcohol. The squares represent the mean OR from each reference dataset (Table S4), horizontal error bars represent the upper and lower 90% CI, the size variation of the boxes represents the weight each dataset had in the meta-analysis with a larger size indicating larger weights. The diamond and dashed line represent the summary effect OR based on the meta-analysis. The ‘line of no effect’ at an OR of 1 is indicated with a dotted line.

The prenatal alcohol data were composed of six studies that included 30 sampling events [76,84,92,98,99,100]. The original studies investigated the relationship between childhood ADHD, CD, and collective externalizing behavioral outcomes and prenatal exposures to alcohol in participants aged 3−10 years (Table S4). Alcohol exposures were determined by maternal survey data, which collected information regarding the amount of alcohol consumed per day, trimesters in which alcohol was consumed, and number of days during pregnancy when alcohol was consumed. All studies pertaining to prenatal alcohol exposure were analyzed together. The summary effect OR for the relationship between prenatal exposure to alcohol and the incidence of childhood externalizing behavioral outcomes was statistically significant at 1.70 (90%_CI_ 1.33−2.07, *p* < 0.001) with considerable heterogeneity (*T^2^* = 1.0, *I^2^* = 82%) (Table 1, Figure 4). 

A meta-regression was conducted to examine potential sources of heterogeneity in the data, using each externalizing behavioral health outcome and number of participants provided by the original studies. The variables that were observed to be statistically significant in the meta-regression model were the externalizing behavioral outcomes: complete ADHD (*p* < 0.001), hyperactivity (*p* = 0.001), inattention (*p* = 0.004), CD/ODD (*p* = 0.003), and the number of participants in the original study (*p* = 0.004). The collective ‘externalizing behaviors’ as an outcome was not significant in this model. The number of significantly influential variables resulted in a reduction in overall heterogeneity (*T*^2^*_Reg_* = 0.5, *I^2^_Reg_* = 66% from *T*^2^*_RMA_* = 1.0, *I*^2^*_RMA_* = 82%), with 48% of the *I*^2^*_Reg_* value being accounted for by the regression model. Even though there was variance in the original data, the evidence suggests prenatal exposure to alcohol may lead to a slight increase in risk for developing externalizing behavioral health outcomes in childhood (Table 1).

#### 3.2.4. Marijuana, Methamphetamine, and Pharmaceuticals

The screening process provided one reference that examined prenatal exposure to marijuana, one reference that examined prenatal exposure to methamphetamine, and one reference that examined prenatal exposure to an antidepressant [76,101,102]. Due to the low number of references in each of these groups, a meta-analysis could not be conducted.

Barthelemy et al. [76] observed that a small percentage of their participants were prenatally exposed to marijuana and measured the effect of prenatal marijuana exposure on the development of externalizing behavioral health outcomes by comparing exposed to non-exposed participants. The OR was 4.26 (95%_CI_ 3.60−12.1), which suggests a relationship between prenatal marijuana exposure and externalizing behaviors, but there was a wide range of variation in the participants’ results [76]. 

Diaz et al. [101] examined the relationship between prenatal methamphetamine exposure and childhood externalizing outcomes. The specific diagnoses examined were ODD and ADHD, which were found to have ORs of 1.35 (95%_CI_ 0.6−3.0) and 1.46 (0.6−3.4), respectively. While the ORs from these comparisons suggest a relationship, the imprecision of the confidence intervals means that a causal relationship cannot be speculated [101].

Brandlistuen et al. [102] investigated the relationship between prenatal antidepressant exposure and the development of childhood externalizing behavioral health outcomes for participants 18 months and 36 months of age. The ORs for externalizing behavioral health outcomes were 1.30 (95%_CI_ 0.95−1.75) and 0.92 (95%_CI_ 0.64−1.31), respectively, which suggests a relationship, but more research must be conducted prior to a conclusion being drawn [102]. 

### 3.3. Environmental Chemical Exposures

#### 3.3.1. Phthalates/Plasticizers (PhPl)

The PhPl data were extracted from four references totaling 79 sampling events with participants ranging in age from 2−18 years old [20,22,23,103]. Since phthalates are known to affect males and females differently through their action on the endocrine system, the sex-specific data examined by the original studies were analyzed separately from the studies that reported combined-sex data. The RMA meta-analytic comparison of the combined-sex data and externalizing behavioral health outcomes resulted in a non-significant summary effect OR of 1.13 (90%_CI_ 0.98−1.27, *p* < 0.0001) with negligible heterogeneity (*T*^2^ = 0, *I*^2^ = 0%). The summary effect OR for the combined-sex data suggests that PhPl exposure may increase externalizing behavioral health outcomes slightly, but the data are variable (Table 1, Figure S2).

**Figure 5:**
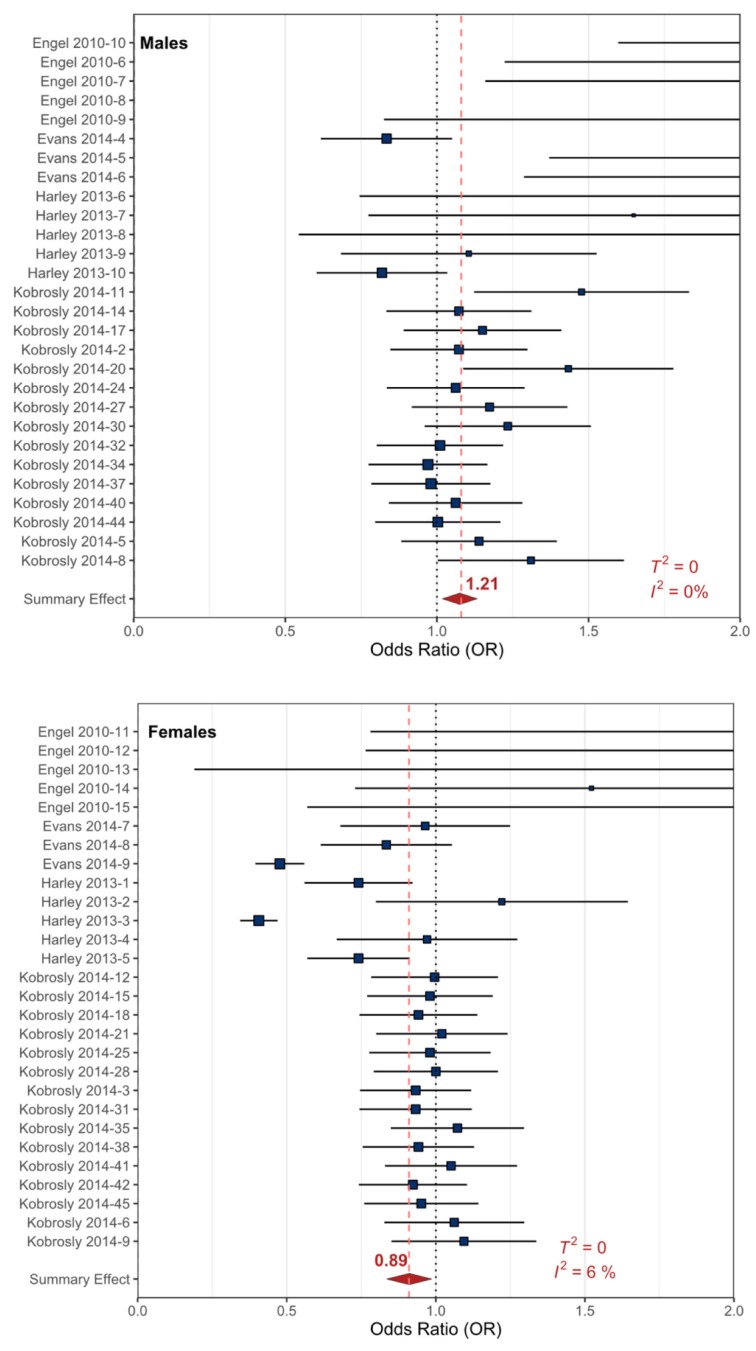
Forest plots showing the externalizing behavioral health outcomes included in the random effects meta-analysis for prenatal PhPl exposure. The squares represent the mean OR from each reference dataset (Table S5), horizontal error bars represent the upper and lower 90% CI, the size variation of the boxes represents the weight each dataset had in the meta-analysis with a larger size indicating larger weights. The diamond and dashed line represent the summary effect OR based on the meta-analysis. The ‘line of no effect’ at an OR of 1 is indicated with a dotted line.

The RMA meta-analysis of the male-specific data and related externalizing behavioral health outcomes consisted of 28 sampling events from four studies and resulted in a statistically significant OR of 1.21 (90%_CI_ 1.06–1.38, *p* < 0.001) with negligible heterogeneity (*T^2^* = 0, *I^2^* = 0%) (Figure 5). The summary effect OR suggests that there is a relationship between prenatal PhPl exposures and externalizing behavioral health outcomes. The RMA meta-analytic comparison of the female-specific data and externalizing behavioral health outcomes consisted of 28 sampling events from four studies and resulted in a statistically significant OR of 0.89 (90%_CI_ 0.77–1.00, *p* < 0.001), with moderate to negligible heterogeneity (*T^2^* = 0, *I^2^* = 6%) (Figure 5). Interestingly, this female-specific OR suggests the opposite of the male-specific OR, in that according to these data, female prenatal PhPl exposure may not be directly related to the development of externalizing behavioral health outcomes (Table 1). The data suggest that prenatal PhPl exposures may result in slight increases in externalizing behaviors in male children, but on the contrary, prenatal PhPl exposures may not be directly related to externalizing behavioral outcomes in female children (Table 1). 

#### 3.3.2. Organic Contaminants (OCs)

The prenatal organic contaminant data was extracted from eight references and were composed of 37 sampling events [104,105,106,107,108,109,110,111]. The included studies investigated the relationship between a suite of OCs and childhood externalizing behavioral health outcomes collectively, as well as specific ADHD behavioral health outcomes in children aged 2–12 years. When all externalizing behavioral health outcomes were pooled the summary effects OR was 0.91 (90%_CI_ 0.78–1.04, *p* < 0.001), with moderate heterogeneity (*T^2^* = 0.1 *I^2^* = 54%) (Table 1, Figure S3). The ADHD-specific prenatal exposures to OCs data were examined using the RMA meta-analysis and resulted in an OR of 0.99 (90%_CI_ 0.87–1.11, *p* < 0.001) and moderate heterogeneity in the included studies (*T^2^* = 0, *I^2^* = 12%) (Table 1). The data analyzed here suggest that prenatal exposure to OCs could increase the risk for developing ADHD and externalizing behavioral health outcomes, but a definitive conclusion is not ascertainable based on these data (Table 1).

#### 3.3.3. Mercury (Hg)

The included prenatal Hg data were extracted from three references, totaling 20 sampling events [81,109,112]. The included studies examined the relationship between prenatal Hg exposure and the development of ADHD, ODD/CD, and collective externalizing behavioral health outcomes in children aged 8–14 years. A meta-analysis for all externalizing behavioral health outcomes was conducted. Additionally, there were enough ADHD-specific data to examine ADHD outcomes separately (Table S7). 

**Figure 6:**
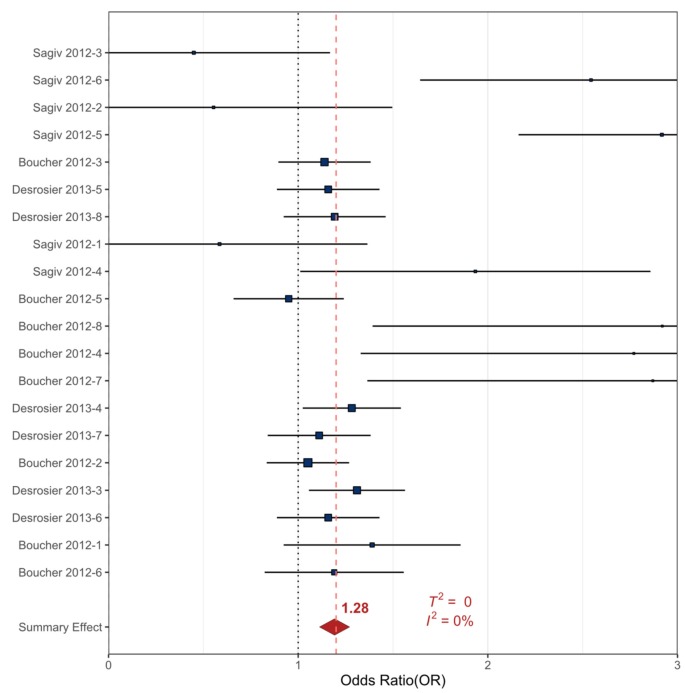
Forest plot showing the random effects meta-analysis for prenatal exposure to Hg and all externalizing behavioral health outcomes. The squares represent the mean OR from each reference datum (Table S6), horizontal error bars represent the upper and lower 90% CI, the size variation of the boxes represent the weight each dataset had in the meta-analysis with a larger size indicating larger weights. The diamond and dashed line represent the summary effect OR based on the meta-analysis. The ‘line of no effect’ at an OR of 1 is indicated with a dotted line.

The summary OR for all externalizing outcomes and prenatal Hg exposure was statistically significant at 1.28 (90%_CI_ 1.10–1.46, *p* < 0.001) and had negligible heterogeneity (*T^2^* = 0, *I^2^* = 0%) (Table 1, Figure 6). The summary OR for the prenatal Hg exposure data and ADHD-specific behavioral health outcomes consisted of 13 sampling events from three studies and was statistically significant at 1.49 (90%_CI_ 1.12–1.86, *p* < 0.001), with greater heterogeneity than the combined outcomes analysis (*T^2^* = 0.27, *I^2^* = 45%) (Table 1). The data analyzed here suggest that prenatal Hg exposure may slightly increase the risk for developing ADHD and externalizing behavioral health outcomes in children (Table 1, Table S7, Figure 6).

#### 3.3.4. Lead (Pb)

The search and inclusion criteria yielded one reference that examined prenatal Pb exposure and externalizing behavioral health outcomes in children. Boucher et al. [110] examined the effect of prenatal Pb exposure on developing ADHD and externalizing behavioral health outcomes in children aged 8–14 years. The OR observed for all externalizing behaviors as a result of prenatal Pb exposure was 1.1 (95%_CI_ 1.0–1.26) and 1.05 (0.90–1.21) for ADHD behavioral health outcomes [110]. While both ORs indicate an increased risk for developing externalizing behaviors following prenatal Pb exposure, these relationships must be further validated to draw a conclusion. 

### 3.4. Non-Chemical Stressor Data

There were six references that examined the relationship between prenatal exposure to non-chemical stressors (e.g., traumatic/stressful experiences), chemical stressors, and childhood behavioral outcomes [76,77,86,113,114,115]. Three references examined maternal trauma/stress during pregnancy and three examined prenatal trauma/stress exposure.

#### 3.4.1. Maternal Stress during Pregnancy

Maughan et al. [86] reported that maternal antisocial behavior, depression, socioeconomic disadvantage, and family adversities were significantly correlated with maternal smoking during pregnancy. In the examined cohort, approximately 75% of childhood conduct problems related to maternal smoking during pregnancy could be accounted for by these non-chemical stressors [86]. Cornelius et al. [113] reported that maternal depression, hostility, and anxiety were significant covariates when linked to prenatal exposure to cigarette smoke and resulted in children who had higher rates of delinquent, aggressive, and externalizing behavioral health outcomes when compared to non-exposed children [113]. Thapar et al. [115] observed the opposite for childhood ADHD symptoms; when genetics, environmental factors, and maternal smoking during pregnancy were combined, there was a significant association with ADHD behavioral health outcomes. However, when maternal smoking during pregnancy was removed from the model, it was no longer statistically significant in the examined cohort. Taken together, these three studies suggest that maternal mental health during pregnancy and subsequent stress-relieving behaviors may contribute to the development of childhood externalizing behavioral health outcomes.

#### 3.4.2. Exposure to Prenatal Stress

Prenatal cocaine exposure was related to greater inattention and externalizing behavioral health outcomes in adolescents compared to non-exposed participants [114]. Min et al. [114] also reported that prenatal exposure to marijuana, family conflict, and exposure to violence were predictors of adolescent externalizing behavior. When prenatal cocaine exposure was coupled with the stress of living in foster care, the incidence of externalizing behavioral health outcomes increased compared to those prenatally exposed and living with their biological parents [114]. Barthelemy et al. [76] observed that the interaction of prenatal exposures to recreational drugs and violence was not correlated with aggressive behavior, but a child’s postnatal exposure was significantly correlated to aggressive behavior at ages 8–11 years. Boden et al. [77] examined the combined effects of all factors and observed that the net contribution of childhood exposure to violence, parental maladaptive behaviors, deviant peers, and maternal smoking during pregnancy contributed to CD and/or ODD behavioral health outcomes. These studies suggest that childhood exposures to traumatic experiences are related to externalizing behavioral health outcomes, but the direct effect of each stressor varies in relation to specific diagnoses. 

### 3.5. MAOA Data

Two studies included in this review examined the relationship between MAOA characteristics and exposures to chemical stressors. Abdelouahab et al. [116] examined the relationship between maternal and umbilical cord metal concentrations and placental MAOA expression of MAOA-L (low activity) and MAOA-H (high activity) genotypes. They observed that placental MAOA enzymatic activity was significantly correlated to maternal blood manganese (Mn) measurements, as well as cord blood Pb, Mn, and cadmium (Cd) measurements. When MAOA-L and MAOA-H genotypes were examined separately, they observed that MAOA-L was significantly correlated with Mn exposures measured in maternal and cord blood samples. The MAOA-H genotype was observed to be significantly correlated with maternal hair Hg measurements. 

Wakschlag et al. [89] examined the interactions between prenatal exposure to cigarette smoke, MAOA genotype, and childhood behavior. They observed that CD symptoms were significantly correlated with the MAOA genotype and prenatal exposure to cigarette smoke, both singularly and as an interactive effect in both male and female adolescents. They also reported that MAOA-L males and MAOA-H females were more likely to have CD symptoms than their counterparts. 

## 4. Discussion

**Figure 7:**
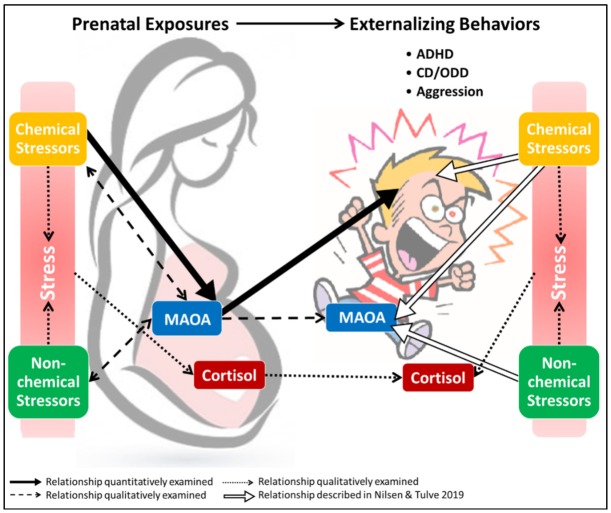
Visual representation depicting the interrelationships between prenatal exposure to chemical and non-chemical stressors, MAOA, cortisol, and their collective influence on children’s externalizing outcomes.

The data evaluated in this review highlight the relationships between prenatal exposures to chemical and non-chemical stressors, MAOA characteristics, and externalizing behaviors in children (Figure 7). The results from the chemical stressor meta-analyses support the idea that prenatal exposure to chemicals can increase the risk of developing externalizing behavioral health outcomes in children (Figure 7, blue arrow). The qualitative analysis of the MAOA data highlights the complexities of genetic predisposition combined with prenatal exposures to chemical stressors (Figure 7, dashed lines). Non-chemical stressors, specifically traumatic and stressful experiences, affect childhood behavioral outcomes differently if they were experienced by the mother during pregnancy or directly by the child either prenatally or in early childhood. Maternal stress manifests by altering cortisol concentrations and when the cortisol concentration increases during pregnancy, the effects are reflected in the child’s altered behavioral outcomes and their stress response (Figure 7, dashed lines). When trauma is experienced directly by the child, it affects their behavior and the type of response they exhibit can be influenced by their MAOA genotype (Figure 7, dashed lines).

The discussion that follows describes the interactions of chemical and non-chemical stressors and MAOA genotypes observed in this review. Further, we explore how maternal cortisol concentrations could be utilized to describe prenatal non-chemical stressor exposures. We discuss the potential use of maternal cortisol levels as a biomarker for exposure to non-chemical stressors, and the potential for cortisol measures to elucidate the relationship between exposures to chemical and non-chemical stressors.

### 4.1. Exposures to Chemical Stressors

The data examined in this review support a relationship between prenatal exposures to chemical stressors and an increased risk for developing externalizing behavioral health outcomes in children (Table 1, Figure 7). The data examined were separated into two categories: prenatal exposures to recreational drugs and environmental chemicals.

#### 4.1.1. Exposure to Recreational Drugs

All prenatal recreational drug data examined (cigarette smoke, cocaine, and alcohol) resulted in statistically significant relationships in the meta-analyses despite the heterogeneity of the included data (Table 1, Figure 2, Figure 3 and Figure 4). The heterogeneity between datasets likely stems from the variation of methodologies used in the original studies. Survey questions regarding the amount of drug use varied and quantified either volumetric estimates of consumption, number of units used, or described use as “light “, “frequent”, or “heavy” to determine prenatal exposure. There were also variations in how the timing of exposure was recorded (i.e., trimester of use or any use during pregnancy) (Table S2). 

Annually, 375,000 newborns are prenatally exposed to recreational drug use in the United States, eliciting a well-documented public health concern [117]. It is important to note that prenatal exposure to drugs does not occur in a vacuum that is void of other stressors. The childhood well-being framework may aid in the identification of other stressors that can be related to prenatal exposure to recreational drugs [44]. A previous study examined contributing factors from the built, natural, and social environments that may be linked to prenatal exposure to recreational drugs [118]. Parental lifestyle factors, part of the social environment, are directly related to parental drug use during pregnancy. Maternal mental health status, which is considered an inherent characteristic, can also be linked to prenatal drug exposures, particularly if the mother is prescribed mood-altering medications. The influence from both the social environment and inherent characteristics in this context can lead to problematic birth outcomes [8,9,12]. The interconnected factors highlighted in this study elucidate the complexity of prenatal exposure to drugs, and additional unexamined covariates could explain some of the variation observed in our study. 

#### 4.1.2. Exposure to Environmental Contaminants

The environmental contaminant data examined in this review were separated into different classes: PhPls, OCs, and Hg. The PhPl data yielded statistically significant summary ORs for all externalizing behavioral health outcomes when the sexes were both separated and combined (Figure 5, Figure S2). Only male-specific PhPl data had statistically significant summary CIs, likely due to the large effect of the exposure as well as the greater incidence of externalizing behavioral health outcomes in males (OR = 1.21, Table 1). Sex-specific differences related to PhPl exposure are well documented in the literature [119,120]. PhPls affect the endocrine system and interact directly with the androgen and estrogen receptors [121]. PhPls are known to affect males more drastically than females, and there have been documented physiological and morphological effects [122]. 

The OC data examined in this review yielded summary effect ORs that were not statistically significant but both meta-analyses had CIs that crossed the line of no effect, which makes the observed relationship tenuous. The lack of statistical significance was likely due to the heterogeneity in the OC exposure studies, which included various methodological differences, such as the sample matrix and classes of OCs (Table S6, Figure S3). 

The prenatal Hg exposures examined in this review yielded statistically significant summary ORs for ADHD and all externalizing behavioral health outcomes combined (Figure 6). Exposure to Hg is known to result in neurotoxic effects, with cognitive and motor ability impairment well-documented as an effect of exposure [123]. The association between Hg exposures and negative mental health outcomes is emerging as a topic in the literature [124,125]. Some of the highest childhood Hg exposures occur in subsistence hunting populations where native cultural paradigms take precedence over modern customs [110]. Subsistence hunting cultures frequently consume long-lived marine predators that are known to have high concentrations of Hg and other pollutants in their tissues [126,127,128]. Pregnant women and those of child-bearing age have been documented to have dietary deficiencies in folate and other vitamins necessary for healthy embryonic development and traditional subsistence foods make up a large portion of their diet [129].

Due to the prominence of cultural practices within society and the often-stark contrast with modern/Western customs, subsistence hunting populations are affected by a variety of non-chemical stressors that are not experienced elsewhere [130]. The loss of cultural identity creates constant stress as more of the population’s activities and behaviors shift to modern practices [131]. There is also a significant stress created by the influence of recreational drugs and a high incidence of violence against women in these populations [43,132]. The perceived negative stigma of mental illness among Native American and Native Alaskan nations can also influence the intervention and treatment measures received [133]. These non-chemical stressors from the social environment may have additional or synergistic effects on mental health outcomes when combined with chemical stressors. The association with prenatal Hg exposure and non-chemical stressors has not been assessed in the literature and could be an interesting topic for future studies. 

### 4.2. MAOA

The MAOA studies included in this review examined the MAOA-L and MAOA-H genotypes in relation to exposure to chemical stressors. The studies reported genotype-based differences in susceptibility to externalizing behaviors [89,116]. The MAOA-H genotype was reported to be sensitive to exposure to both maternal Hg and cigarette smoke, and MAOA-H female children were more likely to be diagnosed with CD after each of the exposures [89,116]. The MAOA-L genotype was reported to be sensitive to exposure to maternal Pb, Mn, Cd, and cigarette smoke, but MAOA-L males were likely to have CD symptoms as a result of exposure [89,116]. 

The observed genotype discrepancies are likely indicative of more specific and/or persistent effects of Hg and cigarette smoke on the MAOA enzyme eliciting MAOA-H (which produces a “normal” amount of the MAOA enzyme to process serotonin) to respond similar to MAOA-L (which produces less of the MAOA enzyme) [134,135]. Sex-based differences have also been previously reported. The female MAOA genotype is more complicated to discern than the male genotype because MAOA is an X-linked gene, meaning that females have two copies. The dominant copy cannot be determined unless a DNA methylation assay is conducted to identify the silent copy [136,137,138]. However, many studies using female participants do not take this approach, instead the studies classify females by the heterozygosity of their alleles based on known numbers of repeated sequences that are indicative of MAOA-L and MAOA-H. Females also report a greater number of stressful life events than males, which may contribute to the lack of consensus for females in the literature and may have influenced the sex-based differences observed [139,140,141]. 

There were no studies that examined the maternal MAOA genotype in relation to the child’s MAOA genotype that met the inclusion criteria for this review. MAOA genetic characteristics are hereditary and have been the subject of studies investigating the ‘cycle of violence’ in maltreated children [32,34,35]. There appears to be a lack of studies in the literature devoted to female and maternal MAOA inheritance; the current literature focuses on males with female subjects often being a lesser focus perhaps due to the complexity of their genotype [32,142,143,144,145]. No studies investigating the maternal genotype, exposure-induced epigenetic changes, and multigenerational implications were identified by this review. Future research evaluating genetic and epigenetic MAOA inheritance in females may help explain the sex-specific differences that are often observed in the literature and supported by the observations in this review. 

### 4.3. Exposure to Non-Chemical Stressors

The non-chemical stressor exposures examined in this review focused on early childhood and maternal traumatic experiences. The included studies examined exposure to violence, family instability, parental maladaptive behavior, and parental mental health status [76,77,86,113,114,115]. However, the included studies all reported slightly different and, in some cases, conflicting results. Differences in conclusions could be due to the specific parameters examined within each study, as well as the different cohorts examined. Another plausible explanation is that the effects of exposure to non-chemical stressors during pregnancy are complex, particularly when examined as an interacting factor with exposure to other stressors. Previous studies have reported that high maternal stress during pregnancy and certain maternal factors (e.g., depression, drug use) are associated with poor pregnancy outcomes [146]. There is increasing support for the idea that compromised fetal development has health effects that persist into adulthood, leading to an increased risk for deleterious health outcomes throughout their life course [5,6,95]. 

Studies that investigate pregnancy outcomes associated with maternal psychosocial stress usually report physical outcomes, such as low birth weight, reduced head circumference, and pre-term birth [95]. Davis and Sandman [9] reported that elevated levels of maternal cortisol can affect cognitive and motor development in children 12 months postpartum and that pregnancy-specific anxiety had adverse developmental effects. No studies in this review examined the effects of maternal traumatic stress on childhood externalizing behavioral health outcomes. Future studies could focus on this knowledge gap to link gestational studies with research examining early childhood through adolescence. 

### 4.4. Linking Chemical and Non-Chemical Stressors with Inherent Biological Characteristics

The desire to examine the synergistic effects of chemical and non-chemical stressors with inherent characteristics to gain a more comprehensive understanding of children’s health outcomes is gaining momentum in the literature [44,118,147]. However, there is still a paucity of studies that examine these factors collectively. A possible explanation could be that non-chemical stressors and their effects are difficult to measure, whereas chemical exposures have well established measurement methods. Therefore, the two groups of scientists conducting each type of study likely do not have the same assessment skills. Measuring cortisol during a stressful event could be a potential measurement technique that would ascribe a value to some non-chemical stressors (particularly trauma-related stressors). Below, we discuss connecting chemical and non-chemical stressors through cortisol, the relationship between cortisol and MAOA, and the relative impact this approach could have on this body of research.

#### 4.4.1. Non-Chemical Stressors and Cortisol

When women experience physical and psychological intimate partner violence, they have a greater incidence of post-traumatic stress disorder (PTSD) and increased cortisol levels, particularly in the evening when the natural cortisol cycle should be declining to promote rest [148,149]. Approximately 11% of American women experience violence during their pregnancy [150]. Interestingly, the more frequently the violence or victimization was experienced, the lower the cortisol response became compared to those experiencing violence for the first time [149].

#### 4.4.2. Maternal Stress Transfer

Childhood health outcomes related to increased cortisol vary depending on the timing of exposure. When exposed prenatally, the resulting childhood health effects have been documented as altered emotionality, altered brain region size, increased sensitivity to glucocorticoids (another class of steroid hormone), and altered cortisol concentrations and the associated stress response [49,151,152,153,154]. There is also significant support for the transgenerational inheritance of stress through epigenetic changes from mother to child [154]. The epigenetic transmission of PTSD was associated with biological alterations in the HPA axis, specifically decreased glucocorticoid receptors, which also influence the stress response [155]. 

In childhood, the less emotionally available a mother is after a traumatic experience can skew the child’s concept of emotional security [156]. When children witness interparental violence and/or experience diminished caregiving in the early years of their life (a period of rapid neurodevelopment), their biological stress response is disrupted, which can lead to life-long physical and mental illnesses [156,157,158]. Traumatic (stressful) childhood experiences can elicit a suite of internalizing disorders including depression and when the depression resulting from childhood trauma is combined with PTSD, the child’s cortisol response decreases further than with PTSD alone [47]. 

#### 4.4.3. Cortisol, Drug Use, and MAOA

Apart from traumatic and stressful experiences, exposure to recreational drugs has also been shown to influence maternal cortisol concentrations, infant emotionality, and childhood stress reactivity [159,160,161]. Children prenatally exposed to continuous alcohol use had baseline cortisol concentrations significantly lower than those that had either sporadic or no prenatal exposure to alcohol [160]. Some sex-related differences were also reported. All female toddlers (exposed and unexposed) had a negative association with their cortisol response and their testosterone concentrations. However, only the exposed male toddlers had increased cortisol reactivity, irrespective of their testosterone concentrations [160]. Prenatal cocaine exposure was correlated with a blunted cortisol response in exposed 11-year-old children when they experienced domestic violence compared to unexposed children who also experienced domestic violence [162]. 

An interrelationship between MAOA genotype, prenatal stress, and childhood behavior was reported in infants as young as 5 weeks of age [161]. The MAOA genotype significantly influenced the risk of negative emotionality of infants as a singular variable in combination with either deprivation of resources or prenatal life events [161]. When sex, MAOA genotype, and childhood stress were examined together, MAOA-L males and MAOA-H females were significantly correlated with aggressive behavior—a symptom and precursor of externalizing behavior disorders [140]. 

Prenatal maternal anxiety, marital status, marital satisfaction, and prenatal life events significantly correlated with negative emotionality across both genotypes. In this study, ‘prenatal life events’ were significantly correlated with marital status, psychological abuse, maternal anxiety, and marital satisfaction [161]. All possible combinations of positive and negative life events influenced by marital status, psychological abuse, maternal anxiety, and marital satisfaction can influence maternal cortisol concentrations. Taken together, these studies provide a compelling relationship between maternal stress and cortisol during pregnancy, prenatal drug exposure, and MAOA genotype.

#### 4.4.4. Cortisol and Non-Chemical Stressor Evaluation

Using cortisol as a measure of maternal stress during pregnancy provides a measurable unit to quantify exposure to non-chemical stressors that are otherwise difficult to measure. However, there are a few caveats that complicate the relationship. First, the timing of the stressor during pregnancy, as well as the woman’s perception of it, affect the cortisol response [95]. Natural and anthropogenic disasters have been reported to adversely affect women in their first trimester and their offspring, more than those who experienced stressors later in pregnancy [95]. Secondly, maternal cortisol concentrations may not reflect traumatic experiences accurately since frequent trauma reduces the body’s cortisol response [149]. Decreased cortisol as a response to constant stress may have been advantageous evolutionarily, but a lowered stress response can lead women and children to be less vigilant of dangerous situations in their environment. There has been limited research examining the transmission of stress and trauma during pregnancy, but the risk of susceptibility to PTSD and the epigenetic effects to the HPA axis can be passed on to future generations [163]. 

Since there are several variables associated with the cortisol response, it may not be an ideal singular marker than could be used to assess exposure to non-chemical stressors. However, using cortisol measurement in tandem with survey and other existing metrics for evaluation could create a more comprehensive understanding of prenatal exposures and their effects on childhood behavior. Further research should be conducted to evaluate the relationship between MAOA genotype and cortisol reactivity, since there appears to be a sex-based and genotype-specific relationship in the few studies that have been conducted.

### 4.5. Prenatal vs. Childhood Exposures

The prenatal data examined in this meta-analysis and the childhood data that was previously reported by Nilsen and Tulve [30] both observed that exposure to cigarette smoke is statistically significant when related to childhood behavioral health outcomes. Of the environmental chemical exposures examined in the two datasets, the PhPl (phthalate and plasticizer) exposures were statistically significant in both the prenatal and childhood exposure datasets, with different ORs for each sex, but interestingly enough, the magnitude of the ORs were the inverse of each other between the datasets (Figure 5; Nilsen and Tulve [30]–Figure 3). 

Despite the similarities between the prenatal exposure groups examined in this meta-analysis and the childhood exposure groups examined by Nilsen and Tulve [30], there are interesting differences in the datasets worth mentioning. The prenatal data reviewed in this meta-analysis was reported in the original studies via survey responses resulting in a larger amount of variance when compared to measurement data. On the other hand, the childhood exposure data was reported in the original studies as measurements from biological tissues that provided the variance, sensitivity, and conclusions. Hence, the childhood exposure data is more specific than the prenatal data [30].

### 4.6. Limitations

The considerable lack of MAOA data related to prenatal exposures, particularly when compared to the data examined in the childhood exposure publication, highlights a research gap that could drive future research questions [30]. There were also very few studies that examined the effects of prenatal exposure to marijuana, methamphetamine, and other recreational drugs. In the cases of marijuana and methamphetamine, the research is likely ongoing as marijuana has become more prevalent in society in the last decade, and the methamphetamine epidemic has reached alarming levels in recent years. Understanding the effects of these and other common-use drugs on prenatal development is necessary to fully understand the health risks of prenatal reactional drug exposure. 

## 5. Conclusions

This review sought to examine the relationships between prenatal exposures to chemical and non-chemical stressors, MAOA genotype, and childhood externalizing behaviors. Through the meta-analyses conducted, the relationship between prenatal exposure to chemical stressors (in the form of recreational drugs and environmental contaminants) and externalizing behavioral health outcomes were observed to be statistically significant. The MAOA data examined herein demonstrate that genotype can be linked to childhood behavioral health outcomes and the genotype expressed can be influenced by exposure to chemical stressors. Discussion of the traumatic experience data highlights the biological variability involved in the stress response. When all factors are taken together, they highlight the complex interrelationships between chemical and non-chemical stressors, inherent characteristics, and behavioral outcomes. Further examination of cortisol reactivity in response to these factors, both singularly and in tandem, could aid in a deeper understanding of the interactive effects these factors have on each other and childhood behavioral outcomes.

## Figures and Tables

**Table 1:** Summary of findings from the systematic review of prenatal exposures to chemical stressors related to childhood externalizing behaviors. Table was modified from the GRADEpro GDT software.

Outcomes	Anticipated Absolute Effects		Relative Effect (90% CI)	№ of Participants (Observational Studies)	Precision of the Evidence (GRADE)
	U.S. Population Risk with DSM Criteria	Risk with Chemical Stressor Exposures			
Cigarette Smoke Exposures					
**CD/ODD**	**61 per 1,000**	**145 per 1,000**	**OR 2.61** * (2.08 to 3.14)	56835 (13)	⨁⨁⨁◯ MODERATE
ADHD	110 per 1,000	**254 per 1,000**	**OR 2.76** * (1.44 to 4.07)	61706 (13)	⨁⨁⨁◯ MODERATE
All Externalizing	74 per 1,000	**148 per 1,000**	**OR 2.17** * (1.36 to 2.96)	180001 (21)	⨁⨁⨁◯ MODERATE
**Cocaine Exposures**					
All Externalizing	74 per 1,000	**166 per 1,000**	**OR 2.49** * (1.52 to 3.48)	5694 (3)	⨁⨁⨁◯ MODERATE
**Alcohol Exposures**					
All Externalizing	74 per 1,000	**119 per 1,000**	**OR 1.69** * (1.33 to 2.07)	367471 (5)	⨁⨁⨁◯ MODERATE
**Phthalate/Plasticizer Exposures**					
All Externalizing (males)	101 per 1,000	**120 per 1,000**	**OR 1.21** * (1.06 to 1.35)	2813 (4)	⨁⨁⨁◯ MODERATE
All Externalizing (females)	45 per 1,000	**40 per 1,000**	**OR 0.88** * (0.77 to 1.00)	2886 (4)	⨁⨁⨁◯ MODERATE
All Externalizing (both sexes)	74 per 1,000	**84 per 1,000**	**OR 1.13** * (0.98 to 1.27)	3513 (3)	⨁⨁◯◯ LOW
**Organic Contaminant Exposures**					
ADHD	74 per 1,000	**7** **3** **per 1,000**	**OR 0.99** * (0.87 to 1.11)	17410 (7)	⨁⨁◯◯ LOW
All Externalizing	110 per 1,000	**1** **01** **per 1,000**	**OR 0.91** * (0.78 to 1.04)	18164 (8)	⨁⨁◯◯ LOW
**Mercury Exposures**					
ADHD	110 per 1,000	**155 per 1,000**	**OR 1.49** * (1.04 to 1.93)	3422 (3)	⨁⨁⨁◯ MODERATE
All Externalizing	74 per 1,000	**93 per 1,000**	**OR 1.28** * (1.06 to 1.50)	4574 (3)	⨁⨁⨁◯ MODERATE

**The risk in the examined/exposed group** is based on the assumed risk in the comparison group and the **relative effect** of the exposure. **CI:** Confidence interval; **OR:** Odds ratio; *: statistically significant OR. **GRADE Working Group grades of evidence. High (*reserved for randomized controlled trials and other empirical, non-observational studies*):** We are very confident that the true effect lies close to that of the estimate of the effect. **Moderate:** We are moderately confident in the effect estimate: The true effect is likely to be close to the estimate of the effect, but there is a possibility that it is substantially different. **Low:** Our confidence in the effect estimate is limited: The true effect may be substantially different from the estimate of the effect. **Very low:** We have very little confidence in the effect estimate: The true effect is likely to be substantially different from the estimate of effect.

## Supplementary Materials

The following are available online at https://www.mdpi.com/1660-4601/17/7/2361/s1, Figures S1–S3, Tables S1–S7.

## Appendix A

**Search Terms:**
PubMed:
1. ("Environmental Exposure"[Mesh]) AND "violence"[Mesh]) 2. ("Environmental Exposure"[Mesh]) AND "aggression"[Mesh]) 3. ("Environmental Exposure"[Mesh]) AND "Conduct Disorder"[Mesh]) 4. ("Environmental Exposure"[Mesh]) AND "Attention Deficit and Disruptive Behavior Disorders"[Mesh]) 5. ("Environmental Exposure"[Mesh]) AND "Monoamine Oxidase"[Mesh]) 6. ("Personality Disorders"[Mesh]) AND "Monoamine Oxidase"[Mesh]) 7. ("Violence"[Mesh]) AND "Monoamine Oxidase"[Mesh]) 8. ("Aggression"[Mesh]) AND "Monoamine Oxidase"[Mesh]) 9. ("Conduct Disorder"[Mesh]) AND "Monoamine Oxidase"[Mesh]) 10. ("Attention Deficit and Disruptive Behavior Disorders"[Mesh]) AND "Monoamine Oxidase"[Mesh])
Web of Science:
11. (TS=(environmental exposure AND violence)) 12. (TS=(environmental exposure AND aggression)) 13. (TS=(environmental exposure AND conduct disorder)) 14. (TS=(environmental exposure AND Attention Deficit and Disruptive Behavior Disorders)) 15. (TS=(environmental exposure AND monoamine oxidase)) 16. (TS=(personality disorders AND monoamine oxidase)) 17. (TS=(violence AND monoamine oxidase)) 18. (TS=(aggression AND Monoamine oxidase)) 19. (TS=(conduct disorder AND monoamine oxidase)) 20. (TS=(Attention Deficit and Disruptive Behavior Disorders AND Monoamine Oxidase))

**R Studio Syntax:**
Random-effects meta-analysis: Package: metafor Code: rma(yi=OR, xi=V, method=”REML”. Data=Dat)
Forest Plot: Packages: ggplot2, metaviz Code: viz_forest(x=Dat[1:k,c("OR","V")], method="REML", confidence_level=.9, study_labels=PrenatCigSmAllDat[1: k c("Reference")], summary_label="Summary Effect", xlab="Odds Ratio(OR)", x_limit=c(0:4), summary_col=c("firebrick"), text_size=3)+geom_vline(aes(xintercept=1, color=),lty=3)+geom_vline(aes(xintercept=2.5, color="firebrick"), lty=2) +theme(panel.grid.major.x=NULL, panel.grid.minor.x=NULL)+annotate("text",x=,y=,parse=TRUE, label=as.character(expression(bold("OR value"))),col="firebrick")+ annotate("text", x=,y=, parse=TRUE, label=as.character(expression(italic(T^{2})*" = ,")),col="firebrick") +annotate("text",x=,y=,parse=TRUE,label =as.character(expression(italic(I^{2})*" = %")), col="firebrick")
Meta-Regression: Package: metafor Code: rma(yi=OR, xi=V, mods=~Outcome+n, method=”REML”. Data=NameDat)
Bubble Plot: Package: ggplot2 Code: ggplot(Dat,aes(x = OR, y = n,col= Outcome, label=Outcome))+geom_point(shape = 21)+geom_smooth(lwd=1,lty=2,method=’lm’,color="darkred",fill="lightblue",level=0.9)+geom_text(data=Dat,aes(label=Outcome),size=3,nudge_x=.1)+theme_bw()
Sensitivity Analysis: Package: metafor Code: rma(yi=OR, xi=V, method=”REML” *or* “FE” *or* “HE” *or* “DL” *or* “ PM” *or* “EB” *or* “ML”, data=NameDat)
Publication Bias Package: metafor Code: regtest.default(PrenatHgADHDDat$OR, PrenatHgADHDDat$V, PrenatHgADHDDat$SE, PrenatHgADHDDat$ni, predictor = "sei", "ni" = PrenatHgADHDDat$n, digit= 2, verbose = TRUE, control = list(stepadj=0.5)
