# Supplementary material for: A Systematic Review and Meta-Analysis Investigating the Relationship between Exposures to Chemical and Non-Chemical Stressors during Prenatal Development and Childhood Externalizing Behaviors
Source: Int J Environ Res Public Health. 2020 Mar 31;17(7):2361. doi: 10.3390/ijerph17072361 (PMC7177257; doi:10.3390/ijerph17072361)
Supplement: Supplementary file 1 [file ijerph-17-02361-s001.pdf]

# *A systematic review and meta-analysis investigating the relationship between exposures to chemical and non-chemical stressors during prenatal development and childhood externalizing behaviors*

Frances M. Nilsen[1], Jessica Frank[1], Nicolle S. Tulve[2]

**Supplemental Information**

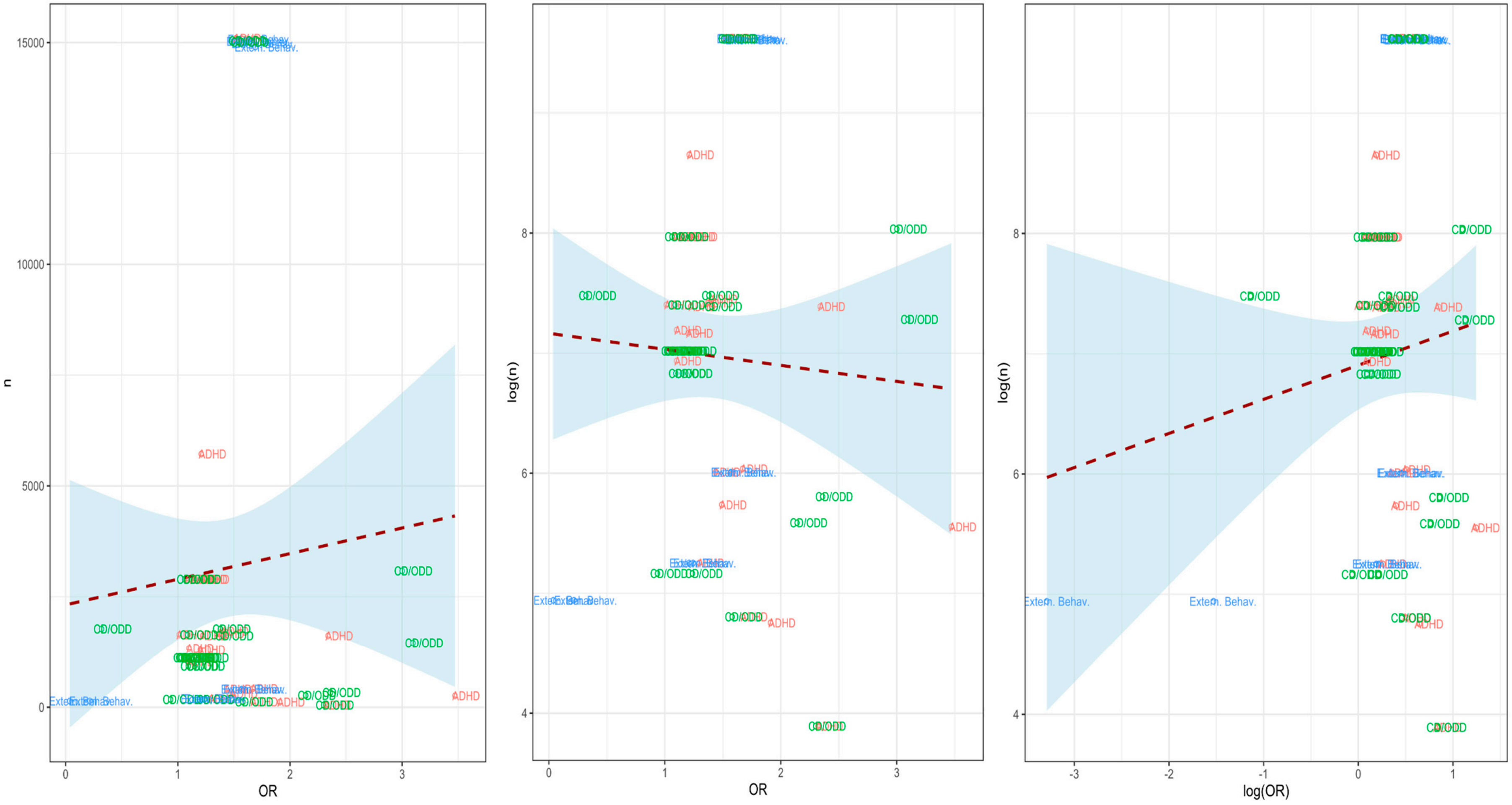


**Figure S1.** The meta-regression plots for the prenatal cigarette smoke exposure and childhood behavioral health outcome data. The odds ratio (OR) is plotted along the x-axis and the number of participants in each study (n) is plotted along the y-axis. The red dashed line represents the regression line and the shaded blue area represents the 95% confidence intervals. Each behavioral health outcome category is represented by a different color and each sampling event is denoted with the behavioral health outcome category text. The behavioral health outcome categories are "ADHD" which includes all ADHD [red text] related outcomes, "CD/ODD" [green text] which includes both conduct and oppositional defiance outcomes, "Extern. Behav." [blue text] represents outcomes that do not fall into the other two categories, as well as the generalized externalizing behavior outcomes that many original studies reported. Plots were made in R Studio using the "*ggplot2"* package.

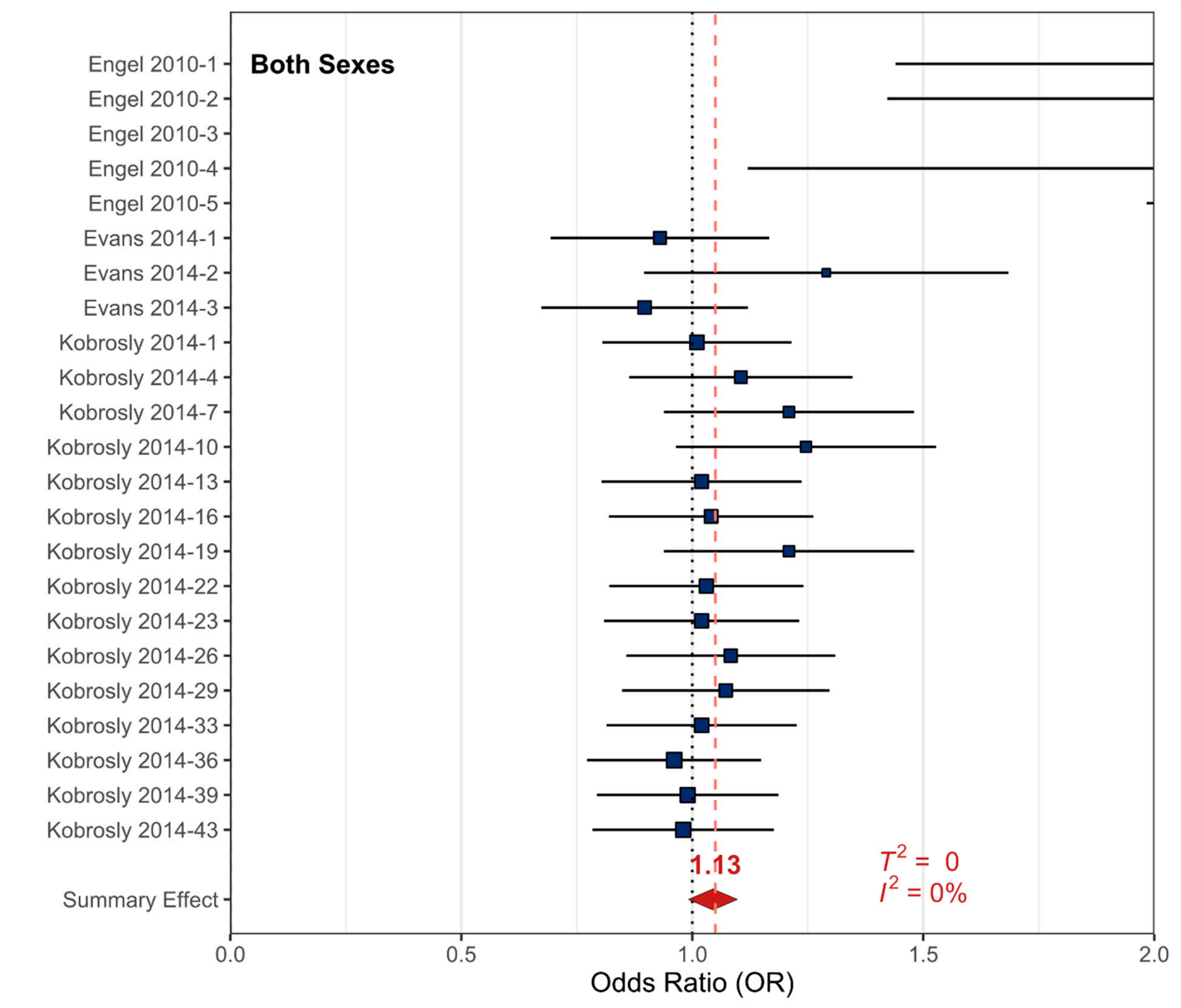


**Figure S2.** Forest plot showing the externalizing behaviors in the random effects meta-analysis for prenatal PhPl exposure (both sexes combined in original studies). The blue squares represent the mean OR from each reference dataset (Table S4), horizontal error bars represent the upper and lower 90% CI, the size variation of the boxes represents the weight each dataset had in the meta-analysis with a larger size indicating larger weights. The red diamond is the summary effect OR based on the meta-analysis. The ‘line of no effect’ at an OR of 1 is indicated with a dotted line.

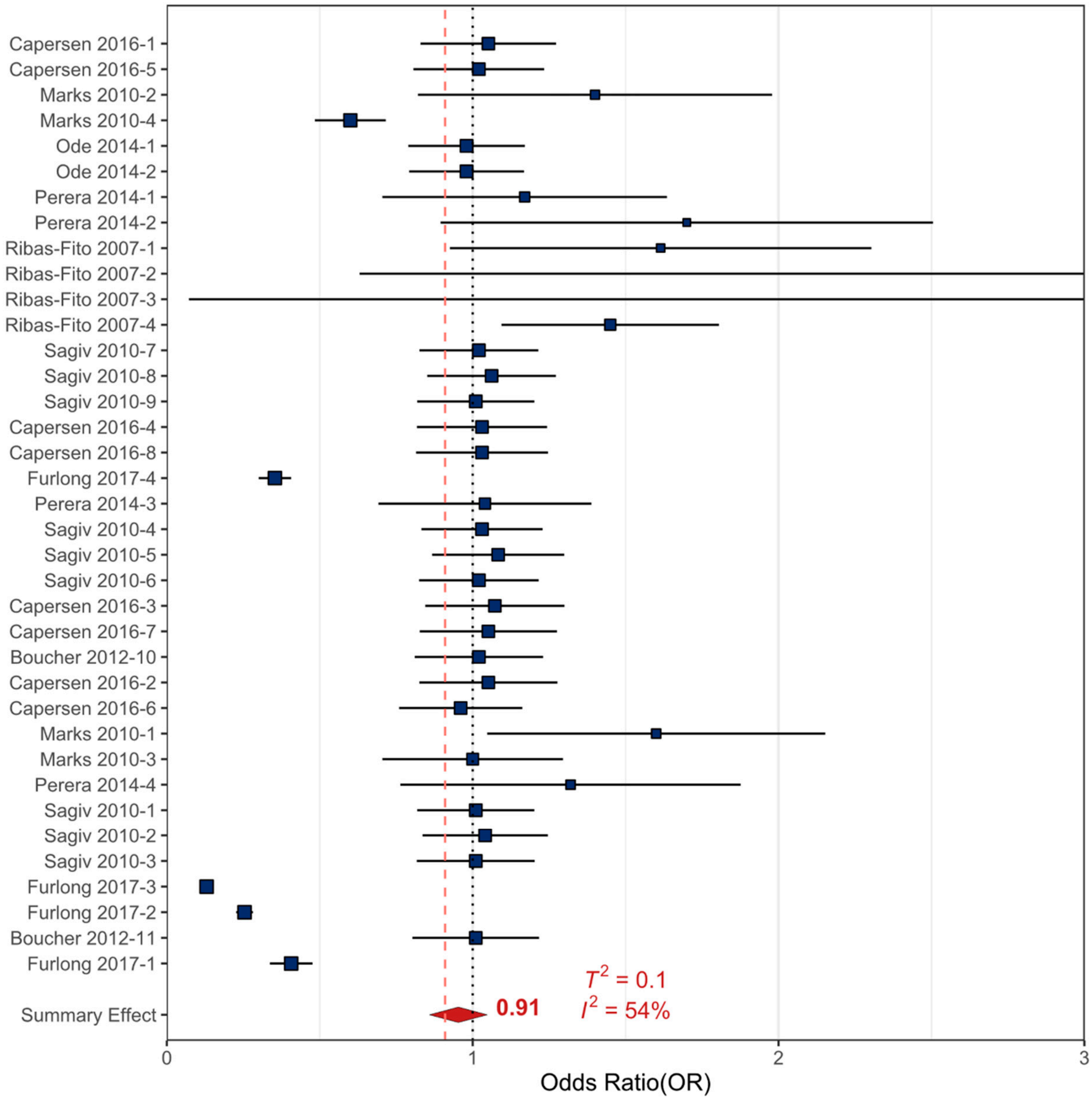


**Figure S3.** Forest plot showing the random effects meta-analysis for prenatal exposure to OCs and all childhood externalizing behavioral health outcomes. The blue squares represent the mean OR from each reference dataset (Table S5), horizontal error bars represent the upper and lower 90% CI, the size variation of the boxes represents the weight each dataset had in the meta-analysis with a larger size indicating larger weights. The red diamond is the summary effect OR based on the meta-analysis. The 'line of no effect' at an OR of 1 is indicated with a dotted line.

**Table S1.** Publication bias and sensitivity analysis.

| Chemical Stressor | Sensitivity Analysis | | | | | | | | Publication Bias | |
|---|---|---|---|---|---|---|---|---|---|---|
| | Meta-Analysis Estimation Model | | | | | | | Data Robustness | Method | *p* value |
| | REML (this study) | FE | HE | DL | PM | EB | ML | *(% ORs within 15%)* | | |
| Cigarette Smoke | **2.16** | 7.89 | 7.89 | 1.85 | 2.49 | 2.45 | 2.17 | ***71%*** | SE | 0.34 |
| | *OR Difference* | 5.73 | 5.73 | -0.31 | 0.33 | 0.29 | 0.01 | | **1/*n*** | **0.65** |
| | *% Difference* | 114% | 114% | 15% | 14% | 13% | 0% | | | |
| Cocaine | **2.49** | 4.83 | 4.83 | 2.37 | 2.58 | 2.58 | 2.51 | ***71%*** | SE | 0.26 |
| | *OR Difference* | 2.34 | 2.34 | -0.12 | 0.09 | 0.09 | 0.02 | | **1/*n*** | **<0.01** |
| | *% Difference* | 64% | 64% | 5% | 4% | 4% | 1% | | | |
| Alcohol | **1.69** | 3.39 | 1.72 | 1.71 | 1.68 | 1.68 | 1.69 | ***86%*** | SE | 0.66 |
| | *OR Difference* | 1.7 | 0.03 | 0.02 | -0.01 | -0.01 | 0 | | **1/*n*** | **<0.01** |
| | *% Difference* | 67% | 2% | 1% | 1% | 1% | 0% | | | |
| Phthalates/Plasticizers (PhPl) Both Sexes | **1.13** | 1.13 | 1.73 | 1.23 | 1.62 | 1.62 | 1.13 | ***57%*** | SE | <0.01 |
| | *OR Difference* | 0 | 0.6 | 0.1 | 0.49 | 0.49 | 0 | | **1/*n*** | **0.13** |
| | *% Difference* | 0% | 42% | 8% | 36% | 36% | 0% | | | |
| Organic Contaminants | **0.91** | 0.52 | 1.05 | 0.96 | 0.90 | 0.90 | 0.91 | ***86%*** | SE | <0.01 |
| | *OR Difference* | -0.39 | 0.14 | 0.05 | -0.01 | -0.01 | 0 | | **1/*n*** | **0.02** |
| | *% Difference* | 55% | 14% | 5% | 1% | 1% | 0% | | | |
| Mercury (Hg) | **1.28** | 1.28 | 1.37 | 1.3 | 1.32 | 1.32 | 1.28 | ***100%*** | SE | <0.01 |
| | *OR Difference* | 0 | 0.09 | 0.02 | 0.04 | 0.04 | 0 | | **1/*n*** | **0.67** |
| | *% Difference* | 0% | 7% | 2% | 3% | 3% | 0% | | | |
| **Data Subsets** | | | | | | | | | | |
| Cigarette Smoke (ADHD only) | **2.76** | 8.73 | 8.73 | 2.40 | 3.41 | 3.41 | 2.79 | ***43%*** | SE | 0.11 |
| | *OR Difference* | 5.97 | 5.97 | -0.36 | 0.65 | 0.65 | 0.03 | | **1/*n*** | **0.76** |
| | *% Difference* | 104% | 104% | 14% | 21% | 21% | 1% | | | |
| Cigarette Smoke (CD/ODD Only) | **2.61** | 2.62 | 2.62 | 2.62 | 2.62 | 2.62 | 2.62 | ***100%*** | SE | 0.35 |
| | *OR Difference* | 0.01 | 0.01 | 0.01 | 0.01 | 0.01 | 0.01 | | **1/*n*** | **0.64** |
| | *% Difference* | 0% | 0% | 0% | 0% | 0% | 0% | | | |
| Cigarette Smoke (1st Trimester Only) | **1.45** | 1.45 | 1.45 | 1.45 | 1.45 | 1.45 | 1.45 | ***100%*** | SE | 0.97 |
| | *OR Difference* | 0 | 0 | 0 | 0 | 0 | 0 | | **1/*n*** | **0.87** |
| | *% Difference* | 0% | 0% | 0% | 0% | 0% | 0% | | | |
| Phthalates/Plasticizers (PhPl) Males | **1.24** | 1.24 | 2.34 | 1.51 | 2.14 | 2.14 | 1.24 | ***43%*** | SE | <0.01 |
| | *OR Difference* | 0 | 1.1 | 0.27 | 0.9 | 0.9 | 0 | | **1/*n*** | **0.02** |
| | *% Difference* | 0% | 61% | 20% | 53% | 53% | 0% | | | |
| Phthalates/Plasticizers (PhPl) Females | **0.77** | 0.73 | 0.92 | 0.77 | 0.76 | 0.76 | 0.77 | ***86%*** | SE | <0.01 |
| | *OR Difference* | -0.04 | 0.15 | 0 | -0.01 | -0.01 | 0 | | **1/*n*** | **0.1** |
| | *% Difference* | 5% | 18% | 0% | 1% | 1% | 0% | | | |
| Organic Contaminants (ADHD Only) | **0.99** | 0.96 | 1.14 | 0.96 | 0.96 | 0.96 | 0.99 | ***100%*** | SE | <0.01 |
| | *OR Difference* | -0.03 | 0.15 | -0.03 | -0.03 | -0.03 | 0 | | **1/*n*** | **0.3** |
| | *% Difference* | 3% | 14% | 3% | 3% | 3% | 0% | | | |
| Mercury (Hg - ADHD Only) | **1.49** | 1.37 | 1.53 | 1.49 | 1.52 | 1.52 | 1.46 | ***100%*** | SE | <0.01 |
| | *OR Difference* | -0.12 | 0.04 | 0 | 0.03 | 0.03 | -0.03 | | **1/*n*** | **0.93** |
| | *% Difference* | 8% | 3% | 0% | 2% | 2% | 2% | | | |

**Table S2**. References and prenatal cigarette smoke data used in the meta-analyses in this review.

| **Prenatal Smoke Exposure & Behavior Disorders** | | | | | | | | | | |
|---|---|---|---|---|---|---|---|---|---|---|
| **Reference** | **Exposure Period and Frequency** | **Mental Health Assessment** | **Outcome** | **Age Range (years)** | ***n*** | **OR** | **SE** | **V** | **Lower 95% CI** | **Upper 95% CI** |
| Desrosiers 2013-1 | general prenatal | CBCL | ADHD | 11 | 258 | 3.47 | 2.8 | 0.1 | 1.0 | 12.1 |
| Galera 2011-1 | general prenatal | CBCL | ADHD | 8 | 1665 | 1.37 | 0.2 | 18.2 | 0.98 | 1.9 |
| Galera 2011-2 | general prenatal | CBCL | ADHD | 8 | 1721 | 1.41 | 0.2 | 19.0 | 1.03 | 1.93 |
| Joo 2017-1 | general prenatal | DSM | ADHD | 6-10 | 418 | 1.67 | 0.4 | 6.8 | 1.1 | 2.6 |
| Langley 2012-1 | general prenatal | DSM | ADHD | 7 | 5719 | 1.21 | 0.0 | 461.5 | 1.1 | 1.3 |
| Pineda 2007-1 | general prenatal | DSM | ADHD | 8 | 486 | 8.9 | 19.7 | 0.0 | 1 | 78.4 |
| Tiesler 2011-3 | general prenatal | DSM | ADHD | 10 | 49 | 2.32 | 0.9 | 1.2 | 1.1 | 4.8 |
| Tiesler 2011-4 | general prenatal | DSM | ADHD | 10 | 122 | 1.67 | 0.4 | 5.4 | 1.0 | 2.7 |
| Ball 2010-1 | <1/2 pack/day | DSM | ADHD - Inat | 7 | 1294 | 1.20 | 0.2 | 19.4 | 0.8 | 1.7 |
| Ball 2010-2 | 1/2-1 pack/day | DSM | ADHD - Inat | 7 | 1327 | 1.10 | 0.2 | 21.3 | 0.8 | 1.6 |
| Ball 2010-3 | > full pack/day | DSM | ADHD - Inat | 7 | 1027 | 1.10 | 0.3 | 12.0 | 0.7 | 1.8 |
| Cornelius 2007-2 | general prenatal | CBCL | ADHD - Inat | 6 | 407 | 1.44 | 0.3 | 14.3 | 1.0 | 2.0 |
| Joo 2017-2 | general prenatal | DSM | ADHD - Inat | 6-10 | 310 | 1.49 | 0.4 | 6.0 | 0.9 | 2.5 |
| Knopik 2009 - 5 | 1-10 cig/day | DSM | ADHD - Inat | 3-10 | 2892 | 1.24 | 0.1 | 140.1 | 1.1 | 1.4 |
| Knopik 2009 - 6 | 11+ cig/day | DSM | ADHD - Inat | 3-10 | 2892 | 1.09 | 0.1 | 182.5 | 1.0 | 1.2 |
| Palmer 2016-1 | general prenatal | DSM | ADHD - Inat | 12-23 | 1616 | 2.34 | 0.7 | 1.8 | 1.3 | 4.2 |
| Dolan 2016-3 | general prenatal | CBCL | ADHD -Hyp | 3 | 15100 | 1.52 | 0.1 | 328.4 | 1.4 | 1.6 |
| Dolan 2016-7 | general prenatal | CBCL | ADHD -Hyp | 3 | 15058 | 1.51 | 0.1 | 333.0 | 1.4 | 1.6 |
| Joo 2017-3 | general prenatal | DSM | ADHD -Hyp | 6-10 | 116 | 1.91 | 1.0 | 1.0 | 0.8 | 4.8 |
| Palmer 2016-2 | general prenatal | DSM | ADHD -Hyp | 12-23 | 1616 | 1.22 | 0.3 | 14.5 | 0.8 | 1.8 |
| Knopik 2009 - 3 | 1-10 cig/day | DSM | ADHD -Hyp/Imp | 3-10 | 2892 | 1.22 | 0.1 | 168.4 | 1.1 | 1.4 |
| Knopik 2009 - 4 | 11+ cig/day | DSM | ADHD -Hyp/Imp | 3-10 | 2892 | 1.14 | 0.1 | 218.8 | 1.0 | 1.3 |
| Ruckinger 2010-1 | general prenatal | DSM | ADHD-Hyp | 10 | 1638 | 1.01 | 0.2 | 29.9 | 0.71 | 1.4 |
| Hsieh 2010-2 | general prenatal | CBCL | ADHD-Inat | 2 | 191 | 1.29 | 0.4 | 5.5 | 0.71 | 2.4 |
| Hsieh 2010-3 | general prenatal | CBCL | Extern. Behav | 2 | 191 | 1.21 | 0.4 | 6.3 | 0.66 | 2.2 |
| Boden 2010-1 | general prenatal | DSM | CD | 14-16 | 926 | 1.15 | 0.0 | 471.4 | 1.1 | 1.2 |
| Braun 2008-8 | general prenatal | DSM | CD | 8-15 | 3082 | 3.00 | 1.3 | 0.6 | 1.4 | 6.6 |
| Chastang 2015-1 | general prenatal | DSM | CD | 10-12 | 1771 | 1.38 | 0.8 | 1.6 | 0.5 | 3.7 |
| Chastang 2015-2 | general prenatal | DSM | CD | 10-12 | 1771 | 0.32 | 0.6 | 2.8 | 0.0 | 2.4 |
| Gaysina 2013-1 | general prenatal | DSM | CD | 5-16 | 1452 | 3.10 | 2.1 | 0.2 | 1.0 | 9.4 |
| Knopik 2009 - 1 | 1-10 cig/day | DSM | CD | 3-10 | 2892 | 1.12 | 0.0 | 554.7 | 1.0 | 1.2 |
| Knopik 2009 - 2 | 11+ cig/day | DSM | CD | 3-10 | 2892 | 1.06 | 0.0 | 554.3 | 1.0 | 1.1 |
| Maughan 2004-1 | 1-9 cig/day | CBCL | CD | 5 | 1116 | 1.11 | 0.1 | 260.2 | 0.9 | 1.1 |
| Maughan 2004-2 | 10-14 cig/day | CBCL | CD | 5 | 1116 | 1.06 | 0.0 | 419.5 | 1.0 | 1.2 |
| Maughan 2004-3 | 15+ cig/day | CBCL | CD | 5 | 1116 | 1.09 | 0.1 | 221.8 | 1.0 | 1.2 |
| Maughan 2004-4 | 1-9 cig/day | CBCL | CD | 7 | 1116 | 1.05 | 0.1 | 264.3 | 0.9 | 1.2 |
| Maughan 2004-5 | 10-14 cig/day | CBCL | CD | 7 | 1116 | 1.03 | 0.0 | 445.4 | 0.9 | 1.1 |

| | | | | | | | | | | |
|---|---|---|---|---|---|---|---|---|---|---|
| Maughan 2004-6 | 15+ cig/day | CBCL | CD | 7 | 1116 | 1.19 | 0.1 | 120.6 | 1.0 | 1.4 |
| Palmer 2016-3 | general prenatal | DSM | CD | 12-18 | 1616 | 1.40 | 0.2 | 22.5 | 1.1 | 1.9 |
| Ruckinger 2010-2 | general prenatal | DSM | CD | 10 | 1638 | 1.09 | 0.2 | 24.2 | 0.8 | 1.6 |
| Tiesler 2011-1 | general prenatal | DSM | CD | 10 | 49 | 2.30 | 0.7 | 1.8 | 1.3 | 4.2 |
| Tiesler 2011-2 | general prenatal | DSM | CD | 10 | 122 | 1.58 | 0.3 | 9.0 | 1.1 | 2.4 |
| Wakschlag 2010-2 | general prenatal | DSM | CD (boys) | 15 | 175 | 1.24 | 0.1 | 136.3 | 1.1 | 1.4 |
| Wakschlag 2010-1 | general prenatal | DSM | CD (girls) | 15 | 175 | 0.94 | 0.0 | 985.7 | 0.9 | 1.0 |
| Desrosiers 2013-2 | general prenatal | CBCL | CD/ODD | 11 | 267 | 2.14 | 1.3 | 0.6 | 0.8 | 5.8 |
| McLaughlin 2011-1 | general prenatal | CBCL | CD/ODD | 9 | 332 | 2.36 | 0.2 | 27.3 | 1.0 | 1.8 |
| Barthelemy 2016-1 | prenatal (10+ vs none) | CBCL | Extern. Behav | 10 | 140 | 0.22 | 4.1 | 0.1 | -7.8 | 8.2 |
| Barthelemy 2016-2 | prenatal (1-10 vs none) | CBCL | Extern. Behav | 10 | 140 | 0.04 | 4.4 | 0.1 | -8.7 | 8.7 |
| Cornelius 2007-3 | general prenatal | CBCL | Extern. Behav | 6 | 407 | 1.59 | 0.3 | 11.6 | 1.1 | 2.3 |
| Dolan 2016-2 | general prenatal | CBCL | Extern. Behav | 3 | 15077 | 1.63 | 0.1 | 285.7 | 1.5 | 1.7 |
| Dolan 2016-6 | general prenatal | CBCL | Extern. Behav | 3 | 15030 | 1.62 | 0.1 | 290.0 | 1.5 | 1.7 |
| Cornelius 2007-1 | general prenatal | CBCL | Extern. Behav. | 6 | 407 | 1.57 | 0.3 | 12.0 | 1.1 | 2.2 |
| Dolan 2016-4 | general prenatal | CBCL | Extern. Behav. | 3 | 14976 | 1.66 | 0.1 | 274.7 | 1.5 | 1.8 |
| Dolan 2016-8 | general prenatal | CBCL | Extern. Behav. | 3 | 14901 | 1.69 | 0.1 | 265.4 | 1.6 | 1.8 |
| Hsieh 2010-1 | general prenatal | CBCL | Extern. Behav. | 2 | 191 | 1.24 | 0.4 | 6.0 | 0.7 | 2.3 |
| Boden 2010-2 | general prenatal | DSM | ODD | 14-16 | 926 | 1.09 | 0.0 | 521.0 | 1.0 | 1.2 |
| Dolan 2016-1 | general prenatal | CBCL | ODD | 3 | 15042 | 1.52 | 0.1 | 326.9 | 1.4 | 1.6 |
| Dolan 2016-5 | general prenatal | CBCL | ODD | 3 | 15011 | 1.54 | 0.1 | 318.6 | 1.4 | 1.7 |
| **First Trimester Only Data** | | | | | | | | | | |
| **Reference** | **Exposure Period and Frequency** | **Mental Health Assessment** | **Outcome** | **Age Range (years)** | ***n*** | **OR** | **SE** | **V** | **Lower 95% CI** | **Upper 95% CI** |
| Cornelius 2007-4 | 1st trimester only | CBCL | Extern. Behav. | 6 | 355 | 1.34 | 0.3 | 14.0 | 0.9 | 2.0 |
| Cornelius 2007-5 | 1st trimester only | CBCL | ADHD - Inat | 6 | 355 | 1.41 | 0.3 | 12.7 | 1.0 | 2.1 |
| Cornelius 2007-6 | 1st trimester only | CBCL | Extern. Behav. | 6 | 355 | 1.62 | 0.3 | 9.5 | 1.1 | 2.4 |
| Knopik 2009 - 7 | 1st Trimester; 1-10 cig/day | DSM | ADHD -Hyp/Imp | 3-10 | 2892 | 1.33 | 0.1 | 128.3 | 1.2 | 1.5 |
| Knopik 2009 - 8 | 1st trimester; 11+ cig/day | DSM | CD | 3-10 | 2892 | 1.10 | 0.1 | 235.6 | 1.0 | 1.2 |
| Knopik 2009 - 9 | 1st trimester; 11+ cig/day | DSM | ADHD -Hyp/Imp | 3-10 | 2892 | 1.07 | 0.1 | 141.8 | 0.9 | 1.2 |
| Knopik 2009 - 10 | 1st Trimester; 1-10 cig/day | DSM | ADHD - Inat | 3-10 | 2892 | 1.18 | 0.1 | 150.1 | 1.0 | 1.4 |
| Knopik 2009 - 11 | 1st trimester; 11+ cig/day | DSM | ADHD - Inat | 3-10 | 2892 | 1.11 | 0.1 | 110.9 | 0.9 | 1.3 |
| Knopik 2009 - 12 | 1st Trimester; 1-10 cig/day | DSM | CD | 3-10 | 2892 | 1.15 | 0.0 | 407.8 | 1.1 | 1.3 |
| Palmer 2016-4 | 1st trimester only | DSM | ADHD - Inat | 12-18 | 1616 | 1.38 | 0.9 | 1.2 | 0.5 | 4.0 |
| Palmer 2016-5 | 1st trimester only | DSM | ADHD -Hyp | 12-18 | 1616 | 1.20 | 0.4 | 7.5 | 0.7 | 2.1 |
| Palmer 2016-6 | 1st trimester only | DSM | CD | 12-18 | 1616 | 1.54 | 0.3 | 15.0 | 1.1 | 2.1 |
| Tiesler 2011-5 | 1st trimester only | SDQ | CD | 10 | 70 | 1.57 | 0.4 | 5.1 | 0.9 | 2.7 |
| Tiesler 2011-6 | 1st trimester only | SDQ | ADHD | 10 | 70 | 1.67 | 0.6 | 3.1 | 0.9 | 3.1 |

DSM – Diagnostic and Statistical Manual of Mental Disorders; CBCL – Child Behavior Checklist; SDQ – Strengths and Difficulties Questionnaire; ADHD – Attention Deficient Hyperactivity Disorder; Hyp – hyperactivity; Imp – impulsiveness; Inat – inattention; Extern. Behav. – general externalizing; ODD – Oppositional Defiance Disorder; CD – Conduct Disorder; OR – odds ratio; SE – standard error; V – variance; CI – confidence intervals.

**Table S3**. References and prenatal cocaine data used in the meta-analyses in this review.

| Prenatal Cocaine Exposure & Behavior disorders | | | | | | | | | | |
|---|---|---|---|---|---|---|---|---|---|---|
| **Reference** | **Exposure Period, Frequency, and Matrix** | **Mental Health Assessment** | **Outcome** | **Age Range (years)** | ***n*** | **OR** | **SE** | **V** | **Lower 95% CI** | **Upper 95% CI** |
| Bada 2007-1 | <3x/week in 1st trimester (Some) | CBCL | Extern. Behav. | 3 | 807 | 1.07 | 0.14 | 0.15 | 0.83 | 1.38 |
| Bada 2007-2 | >3x/week in 1st trimester (High) | CBCL | Extern. Behav. | 3 | 517 | 1.78 | 0.36 | 0.98 | 1.21 | 2.61 |
| Bada 2007-3 | <3x/week in 1st trimester (Some) | CBCL | Extern. Behav. | 5 | 642 | 1.54 | 0.20 | 0.32 | 1.19 | 1.99 |
| Bada 2007-4 | >3x/week in 1st trimester (High) | CBCL | Extern. Behav. | 5 | 345 | 1.91 | 0.41 | 1.32 | 1.26 | 2.89 |
| Bada 2007-5 | <3x/week in 1st trimester (Some) | CBCL | Extern. Behav. | 7 | 719 | 1.38 | 0.18 | 0.25 | 1.07 | 1.78 |
| Bada 2007-6 | >3x/week in 1st trimester (High) | CBCL | Extern. Behav. | 7 | 422 | 2.14 | 0.43 | 1.45 | 1.45 | 3.15 |
| Barthelemy 2016-3 | intrauterine cocaine exposure (high v none) | CBCL | Extern. Behav. | 10 | 140 | 6.49 | 6.49 | 0.02 | -6.23 | 19.21 |
| Barthelemy 2016-4 | intrauterine cocaine exposure (light v one) | CBCL | Extern. Behav. | 10 | 140 | 4.10 | 3.82 | 0.07 | -3.39 | 11.58 |
| McLaughlin 2011-2 | cocaine metabolites in maternal/infant urine/infant meconium, maternal admission of use | CBCL | Extern. Behav. | 9 | 329 | 2.03 | 0.42 | 5.54 | 1.36 | 3.03 |
| McLaughlin 2011-3 | cocaine metabolites in maternal/infant urine/infant meconium, maternal admission of use | CBCL | Extern. Behav. | 9 | 329 | 2.09 | 0.44 | 5.19 | 1.40 | 3.12 |
| McLaughlin 2011-4 | cocaine metabolites in maternal/infant urine/infant meconium, maternal admission of use | CBCL | Extern. Behav. | 9 | 329 | 1.77 | 0.37 | 7.41 | 1.19 | 2.63 |
| McLaughlin 2011-5 | cocaine metabolites in maternal/infant urine/infant meconium, maternal admission of use | CBCL | ODD | 9 | 325 | 1.39 | 0.29 | 11.97 | 0.94 | 2.07 |
| McLaughlin 2011-6 | cocaine metabolites in maternal/infant urine/infant meconium, maternal admission of use | CBCL | CD | 9 | 325 | 1.07 | 0.22 | 20.54 | 0.72 | 1.59 |
| McLaughlin 2011-7 | cocaine metabolites in maternal/infant urine/infant meconium, maternal admission of use | CBCL | ADHD | 9 | 325 | 1.17 | 0.24 | 17.08 | 0.79 | 1.74 |

DSM – Diagnostic and Statistical Manual of Mental Disorders; CBCL – Child Behavior Checklist; SDQ – Strengths and Difficulties Questionnaire; CRS – Conner's Rating Scales; ADHD – Attention Deficient Hyperactivity Disorder; Hyp – hyperactivity; Imp – impulsiveness; Inat – inattention; Extern. Behav. – general externalizing; ODD – Oppositional Defiance Disorder; CD – Conduct Disorder; OR – odds ratio; SE – standard error; V – variance; CI – confidence intervals.

**Table S4.** References and prenatal alcohol data used in the meta-analyses in this review.

| Prenatal Alcohol Exposure & Behavior disorders | | | | | | | | | | |
|---|---|---|---|---|---|---|---|---|---|---|
| **Reference** | **Exposure Period and Frequency** | **Mental Health Assessment** | **Outcome** | **Age Range (years)** | ***n*** | **OR** | **SE** | **V** | **Lower 95% CI** | **Upper 95% CI** |
| Barthelemy 2016 - 5 | prenatal (0.5 drinks/day v none) | CBCL | Extern. Behav. | 10 | 140 | 4.90 | 10.70 | 0.01 | -16.06 | 10.71 |
| Barthelemy 2016 - 6 | prenatal (0.5+ drinks/day v none) | CBCL | Extern. Behav. | 10 | 140 | 1.73 | 5.00 | 0.04 | -8.07 | 5.08 |
| Knopik 2009-13 | 1-10 days | DSM | ADHD - Hyp/Inat | 3-10 | 2892 | 0.57 | 1.04 | 0.93 | 0.88 | 1.02 |
| Knopik 2009-14 | 11-35 days | DSM | ADHD - Hyp/Inat | 3-10 | 2892 | 0.44 | 1.11 | 0.81 | 0.75 | 1.14 |
| Knopik 2009-15 | >35 days | DSM | ADHD - Hyp/Inat | 3-10 | 2892 | 1.10 | 1.16 | 0.74 | 0.82 | 1.47 |
| Knopik 2009-16 | some heavy use | DSM | ADHD - Hyp/Inat | 3-10 | 2892 | 1.03 | 1.12 | 0.79 | 0.82 | 1.29 |
| Knopik 2009-17 | frequent heavy use | DSM | ADHD - Hyp/Inat | 3-10 | 2892 | 1.03 | 1.19 | 0.71 | 0.73 | 1.45 |
| Knopik 2009-18 | 1-10 days | DSM | ADHD - Inat | 3-10 | 2892 | 0.53 | 1.04 | 0.92 | 0.86 | 1.02 |
| Knopik 2009-19 | 11-35 days | DSM | ADHD - Inat | 3-10 | 2892 | 0.97 | 1.13 | 0.79 | 0.76 | 1.22 |
| Knopik 2009-20 | >35 days | DSM | ADHD - Inat | 3-10 | 2892 | 1.24 | 1.22 | 0.67 | 0.84 | 1.84 |
| Knopik 2009-21 | some heavy use | DSM | ADHD - Inat | 3-10 | 2892 | 0.88 | 1.13 | 0.79 | 0.69 | 1.11 |
| Knopik 2009-22 | frequent heavy use | DSM | ADHD - Inat | 3-10 | 2892 | 1.70 | 1.29 | 0.60 | 1.03 | 2.83 |
| Knopik 2009-23 | 1-10 days | DSM | CD | 3-10 | 2892 | 1.00 | 1.02 | 0.95 | 0.95 | 1.05 |
| Knopik 2009-24 | 11-35 days | DSM | CD | 3-10 | 2892 | 0.93 | 1.06 | 0.89 | 0.83 | 1.04 |
| Knopik 2009-25 | >35 days | DSM | CD | 3-10 | 2892 | 1.10 | 1.14 | 0.77 | 0.85 | 1.42 |
| Knopik 2009-26 | some heavy use | DSM | CD | 3-10 | 2892 | 0.95 | 1.10 | 0.83 | 0.79 | 1.14 |
| Knopik 2009-27 | frequent heavy use | DSM | CD | 3-10 | 2892 | 1.47 | 1.17 | 0.73 | 1.08 | 2.01 |
| Knudsen 2014-1 | 1st trimester | CBCL | Extern. Behav. | 1.5 | 56682 | 1.48 | 2.19 | 0.21 | 1.28 | 1.71 |
| Knudsen 2014-2 | 2nd trimester | CBCL | Extern. Behav. | 1.5 | 56682 | 1.56 | 2.12 | 0.22 | 1.35 | 1.79 |
| Knudsen 2014-3 | 3rd trimester | CBCL | Extern. Behav. | 1.5 | 56682 | 1.56 | 2.12 | 0.22 | 1.35 | 1.79 |
| Knudsen 2014-4 | 1st trimester | CBCL | Extern. Behav. | 3 | 46759 | 1.53 | 2.14 | 0.22 | 1.32 | 1.77 |
| Knudsen 2014-5 | 2nd trimester | CBCL | Extern. Behav. | 3 | 46759 | 1.59 | 2.09 | 0.23 | 1.37 | 1.83 |
| Knudsen 2014-6 | 3rd trimester | CBCL | Extern. Behav. | 3 | 46759 | 1.6 | 2.09 | 0.23 | 1.38 | 1.85 |
| Pfinder 2012-1 | prenatal | DSM | ADHD- Hyp/Inat | 3-10 | 837 | 2.558 | 1.41 | 0.50 | 1.651 | 3.962 |
| Pfinder 2012-2 | prenatal | DSM | ADHD- Hyp/Inat | 3-10 | 560 | 5.26 | 0.98 | 1.04 | 3.207 | 8.629 |
| Pfinder 2012-3 | prenatal | DSM | ADHD- Hyp/Inat | 3-10 | 837 | 2.11 | 1.53 | 0.43 | 1.271 | 3.505 |
| Pfinder 2012-4 | prenatal | DSM | ADHD- Hyp/Inat | 3-10 | 560 | 3.092 | 1.17 | 0.74 | 1.646 | 5.809 |
| Pineda 2007-2 | prenatal | DSM | ADHD | 8 | 486 | 14.1 | 0.26 | 15 | 1.7 | 114 |
| Sayal 2014-1 | binge drinking during pregnancy (4+ drinks/day) at least once | DSM | CD | 5 | 4183 | 1.05 | 2.76 | 0.13 | 0.94 | 1.16 |
| Sayal 2014-2 | binge drinking during pregnancy (4+ drinks/day) at least once | DSM | ADHD - Hyp/Inat | 5 | 4183 | 1.12 | 2.61 | 0.15 | 0.95 | 1.31 |

DSM – Diagnostic and Statistical Manual of Mental Disorders; CBCL – Child Behavior Checklist; SDQ – Strengths and Difficulties Questionnaire; CRS – Conner's Rating Scales; ADHD – Attention Deficient Hyperactivity Disorder; Hyp – hyperactivity; Imp – impulsiveness; Inat – inattention; Extern. Behav. – general externalizing; ODD – Oppositional Defiance Disorder; CD – Conduct Disorder; OR – odds ratio; SE – standard error; V – variance; CI – confidence intervals.

**Table S5.** References and prenatal phthalate/plasticizer data used in the meta-analyses in this review.

| Prenatal Phthalate/Plasticizer Exposures & Behavior Disorders | | | | | | | | | | | |
|---|---|---|---|---|---|---|---|---|---|---|---|
| **Reference** | **Biological Sample** | **Mental Health Assessment** | **sex** | **Outcome** | **Age Range (years)** | ***n*** | **OR** | **SE** | **V** | **Lower 95% CI** | **Upper 95% CI** |
| Engel 2010-11 | maternal urine increases in LMWP | BASC | F | Extern. Behav | 5-18 | 161 | 2.4 | 1.0 | 1.0 | 0.4 | 12.4 |
| Engel 2010-12 | maternal urine increases in LMWP | BASC | F | ADHD - Inat | 5-18 | 161 | 2.3 | 1.0 | 0.9 | 0.4 | 12.7 |
| Engel 2010-13 | maternal urine increases in LMWP | BASC | F | CD | 5-18 | 149 | 5.5 | 0.6 | 3.2 | 1.1 | 27.4 |
| Engel 2010-14 | maternal urine increases in LMWP | BASC | F | ADHD - Hyp | 5-18 | 161 | 1.5 | 1.4 | 0.5 | 0.2 | 10.0 |
| Engel 2010-15 | maternal urine increases in LMWP | BASC | F | Extern. Behav | 5-18 | 161 | 3.2 | 0.8 | 1.6 | 0.6 | 18.0 |
| Evans 2014-7 | maternal urine BPA | DSM | F | ADHD | 6-10 | 59 | 1.0 | 2.4 | 0.2 | 0.4 | 2.6 |
| Evans 2014-8 | maternal urine BPA | DSM | F | ODD | 6-10 | 59 | 0.8 | 2.7 | 0.1 | 0.3 | 2.2 |
| Evans 2014-9 | maternal urine BPA | DSM | F | CD | 6-10 | 59 | 0.5 | 4.5 | 0.0 | 0.2 | 1.3 |
| Harley 2013-1 | maternal urine BPA | BASC | F | Extern. Behav | 7 | 155 | 0.7 | 3.0 | 0.1 | 0.3 | 2.0 |
| Harley 2013-2 | maternal urine BPA | BASC | F | Extern. Behav | 7 | 155 | 1.2 | 2.0 | 0.3 | 0.4 | 3.0 |
| Harley 2013-3 | maternal urine BPA | BASC | F | CD | 7 | 156 | 0.4 | 5.1 | 0.0 | 0.1 | 1.5 |
| Harley 2013-4 | maternal urine BPA | BASC | F | ADHD - Hyp | 7 | 155 | 1.0 | 2.3 | 0.2 | 0.3 | 2.0 |
| Harley 2013-5 | maternal urine BPA | BASC | F | ADHD - Inat | 7 | 155 | 0.7 | 3.1 | 0.1 | 0.3 | 1.5 |
| Kobrosly 2014-12 | maternal urine MiBP | CBCL | F | CD | 6-10 | 76 | 1.0 | 2.8 | 0.1 | 0.8 | 1.2 |
| Kobrosly 2014-15 | maternal urine MBP | CBCL | F | ADHD | 6-10 | 76 | 1.0 | 2.8 | 0.1 | 0.8 | 1.2 |
| Kobrosly 2014-18 | maternal urine MBP | CBCL | F | ODD | 6-10 | 76 | 0.9 | 2.9 | 0.1 | 0.8 | 1.2 |
| Kobrosly 2014-21 | maternal urine MBP | CBCL | F | CD | 6-10 | 76 | 1.0 | 2.7 | 0.1 | 0.8 | 1.3 |
| Kobrosly 2014-25 | maternal urine MBzP | CBCL | F | ADHD | 6-10 | 76 | 1.0 | 2.8 | 0.1 | 0.8 | 1.2 |
| Kobrosly 2014-28 | maternal urine MBzP | CBCL | F | ODD | 6-10 | 76 | 1.0 | 2.8 | 0.1 | 0.9 | 1.2 |
| Kobrosly 2014-3 | maternal urine DEHP | CBCL | F | CD | 6-10 | 76 | 0.9 | 3.0 | 0.1 | 0.8 | 1.1 |
| Kobrosly 2014-31 | maternal urine MBzP | CBCL | F | CD | 6-10 | 76 | 0.9 | 3.0 | 0.1 | 0.8 | 1.1 |
| Kobrosly 2014-35 | maternal urine MEP | CBCL | F | ADHD | 6-10 | 76 | 1.1 | 2.7 | 0.1 | 1.0 | 1.2 |
| Kobrosly 2014-38 | maternal urine MEP | CBCL | F | ODD | 6-10 | 76 | 0.9 | 3.0 | 0.1 | 0.8 | 1.1 |
| Kobrosly 2014-41 | maternal urine DEHP | CBCL | F | ADHD | 6-10 | 76 | 1.1 | 2.7 | 0.1 | 0.9 | 1.2 |
| Kobrosly 2014-42 | maternal urine MEP | CBCL | F | CD | 6-10 | 76 | 0.9 | 3.0 | 0.1 | 0.8 | 1.0 |
| Kobrosly 2014-45 | maternal urine DEHP | CBCL | F | ODD | 6-10 | 76 | 1.0 | 2.9 | 0.1 | 0.8 | 1.1 |

| | | | | | | | | | | | |
|---|---|---|---|---|---|---|---|---|---|---|---|
| Kobrosly 2014-6 | maternal urine MiBP | CBCL | F | ADHD | 6-10 | 76 | 1.1 | 2.6 | 0.1 | 0.9 | 1.3 |
| Kobrosly 2014-9 | maternal urine MiBP | CBCL | F | ODD | 6-10 | 76 | 1.1 | 2.6 | 0.1 | 0.9 | 1.3 |
| Engel 2010-10 | maternal urine increases in LMWP | BASC | M | Extern. Behav | 5-18 | 161 | 8.0 | 0.5 | 3.9 | 2.1 | 30.6 |
| Engel 2010-6 | maternal urine increases in LMWP | BASC | M | Extern. Behav | 5-18 | 161 | 4.3 | 0.7 | 1.9 | 1.2 | 15.8 |
| Engel 2010-7 | maternal urine increases in LMWP | BASC | M | ADHD - Inat | 5-18 | 161 | 4.7 | 0.7 | 2.2 | 1.2 | 17.8 |
| Engel 2010-8 | maternal urine increases in LMWP | BASC | M | CD | 5-18 | 149 | 16.3 | 0.4 | 6.0 | 4.7 | 56.3 |
| Engel 2010-9 | maternal urine increases in LMWP | BASC | M | ADHD - Hyp | 5-18 | 161 | 4.0 | 0.7 | 1.9 | 0.9 | 17.3 |
| Evans 2014-4 | maternal urine BPA | DSM | M | ADHD | 6-10 | 66 | 0.8 | 2.8 | 0.1 | 0.3 | 2.1 |
| Evans 2014-5 | maternal urine BPA | DSM | M | ODD | 6-10 | 66 | 2.9 | 1.0 | 0.9 | 1.0 | 7.9 |
| Evans 2014-6 | maternal urine BPA | DSM | M | CD | 6-10 | 66 | 2.6 | 1.1 | 0.8 | 1.0 | 6.9 |
| Harley 2013-6 | maternal urine BPA | BASC | M | Extern. Behav | 7 | 133 | 2.0 | 1.1 | 0.8 | 0.3 | 12.2 |
| Harley 2013-7 | maternal urine BPA | BASC | M | Extern. Behav | 7 | 135 | 1.6 | 1.4 | 0.5 | 0.3 | 8.2 |
| Harley 2013-8 | maternal urine BPA | BASC | M | CD | 7 | 133 | 3.0 | 0.8 | 1.5 | 0.5 | 20.1 |
| Harley 2013-9 | maternal urine BPA | BASC | M | ADHD - Hyp | 7 | 133 | 1.1 | 2.0 | 0.3 | 0.2 | 6.0 |
| Harley 2013-10 | maternal urine BPA | BASC | M | ADHD - Inat | 7 | 133 | 0.8 | 2.8 | 0.1 | 0.3 | 2.2 |
| Kobrosly 2014-11 | maternal urine MiBP | CBCL | M | CD | 6-10 | 77 | 1.5 | 2.2 | 0.2 | 1.2 | 1.8 |
| Kobrosly 2014-14 | maternal urine MBP | CBCL | M | ADHD | 6-10 | 77 | 1.1 | 2.6 | 0.1 | 0.9 | 1.3 |
| Kobrosly 2014-17 | maternal urine MBP | CBCL | M | ODD | 6-10 | 77 | 1.2 | 2.5 | 0.2 | 0.9 | 1.4 |
| Kobrosly 2014-2 | maternal urine DEHP | CBCL | M | CD | 6-10 | 77 | 1.1 | 2.7 | 0.1 | 0.9 | 1.2 |
| Kobrosly 2014-20 | maternal urine MBP | CBCL | M | CD | 6-10 | 77 | 1.4 | 2.2 | 0.2 | 1.2 | 1.8 |
| Kobrosly 2014-24 | maternal urine MBzP | CBCL | M | ADHD | 6-10 | 77 | 1.1 | 2.7 | 0.1 | 0.9 | 1.3 |
| Kobrosly 2014-27 | maternal urine MBzP | CBCL | M | ODD | 6-10 | 77 | 1.2 | 2.5 | 0.2 | 1.0 | 1.4 |
| Kobrosly 2014-30 | maternal urine MBzP | CBCL | M | CD | 6-10 | 77 | 1.2 | 2.4 | 0.2 | 1.1 | 1.4 |
| Kobrosly 2014-32 | maternal urine DEHP | CBCL | M | ADHD | 6-10 | 77 | 1.0 | 2.8 | 0.1 | 0.9 | 1.2 |
| Kobrosly 2014-34 | maternal urine MEP | CBCL | M | ADHD | 6-10 | 77 | 1.0 | 2.9 | 0.1 | 0.9 | 1.1 |
| Kobrosly 2014-37 | maternal urine MEP | CBCL | M | ODD | 6-10 | 77 | 1.0 | 2.9 | 0.1 | 0.9 | 1.1 |
| Kobrosly 2014-40 | maternal urine MEP | CBCL | M | CD | 6-10 | 77 | 1.1 | 2.7 | 0.1 | 0.9 | 1.2 |
| Kobrosly 2014-44 | maternal urine DEHP | CBCL | M | ODD | 6-10 | 77 | 1.0 | 2.8 | 0.1 | 0.9 | 1.2 |
| Kobrosly 2014-5 | maternal urine MiBP | CBCL | M | ADHD | 6-10 | 77 | 1.1 | 2.5 | 0.2 | 0.9 | 1.4 |
| Kobrosly 2014-8 | maternal urine MiBP | CBCL | M | ODD | 6-10 | 77 | 1.3 | 2.3 | 0.2 | 1.1 | 1.6 |

| | | | | | | | | | | | |
|---|---|---|---|---|---|---|---|---|---|---|---|
| Engel 2010-1 | maternal urine increases in LMWP | BASC | M+F | Extern. Behav | 5-18 | 171 | 3.5 | 0.9 | 1.2 | 1.2 | 10.4 |
| Engel 2010-2 | maternal urine increases in LMWP | BASC | M+F | ADHD - Inat | 5-18 | 171 | 3.6 | 0.9 | 1.3 | 1.2 | 11.1 |
| Engel 2010-3 | maternal urine increases in LMWP | BASC | M+F | CD | 5-18 | 159 | 11.0 | 0.5 | 3.6 | 3.8 | 31.8 |
| Engel 2010-4 | maternal urine increases in LMWP | BASC | M+F | ADHD - Hyp | 5-18 | 171 | 2.8 | 1.0 | 1.0 | 0.8 | 9.8 |
| Engel 2010-5 | maternal urine increases in LMWP | BASC | M+F | Extern. Behav | 5-18 | 171 | 5.8 | 0.7 | 2.3 | 1.8 | 17.8 |
| Evans 2014-1 | maternal urine BPA | DSM | M+F | ADHD | 6-10 | 125 | 0.9 | 2.6 | 0.1 | 0.5 | 1.8 |
| Evans 2014-2 | maternal urine BPA | DSM | M+F | ODD | 6-10 | 125 | 1.3 | 2.0 | 0.2 | 0.7 | 2.5 |
| Evans 2014-3 | maternal urine BPA | DSM | M+F | CD | 6-10 | 125 | 0.9 | 2.7 | 0.1 | 0.5 | 1.7 |
| Kobrosly 2014-1 | maternal urine DEHP | CBCL | M+F | CD | 6-10 | 153 | 1.0 | 2.8 | 0.1 | 0.9 | 1.1 |
| Kobrosly 2014-10 | maternal urine MiBP | CBCL | M+F | CD | 6-10 | 153 | 1.2 | 2.4 | 0.2 | 1.1 | 1.5 |
| Kobrosly 2014-13 | maternal urine MBP | CBCL | M+F | ADHD | 6-10 | 153 | 1.0 | 2.8 | 0.1 | 0.9 | 1.2 |
| Kobrosly 2014-16 | maternal urine MBP | CBCL | M+F | ODD | 6-10 | 153 | 1.0 | 2.7 | 0.1 | 0.9 | 1.2 |
| Kobrosly 2014-19 | maternal urine MBP | CBCL | M+F | CD | 6-10 | 153 | 1.2 | 2.5 | 0.2 | 1.0 | 1.4 |
| Kobrosly 2014-22 | maternal urine DEHP | CBCL | M+F | ADHD | 6-10 | 153 | 1.0 | 2.8 | 0.1 | 0.9 | 1.2 |
| Kobrosly 2014-23 | maternal urine MBzP | CBCL | M+F | ADHD | 6-10 | 153 | 1.0 | 2.8 | 0.1 | 0.9 | 1.2 |
| Kobrosly 2014-26 | maternal urine MBzP | CBCL | M+F | ODD | 6-10 | 153 | 1.1 | 2.7 | 0.1 | 1.0 | 1.2 |
| Kobrosly 2014-29 | maternal urine MBzP | CBCL | M+F | CD | 6-10 | 153 | 1.1 | 2.7 | 0.1 | 0.9 | 1.2 |
| Kobrosly 2014-33 | maternal urine MEP | CBCL | M+F | ADHD | 6-10 | 153 | 1.0 | 2.8 | 0.1 | 0.9 | 1.1 |
| Kobrosly 2014-36 | maternal urine MEP | CBCL | M+F | ODD | 6-10 | 153 | 1.0 | 3.0 | 0.1 | 0.9 | 1.1 |
| Kobrosly 2014-39 | maternal urine MEP | CBCL | M+F | CD | 6-10 | 153 | 1.0 | 2.9 | 0.1 | 0.9 | 1.1 |
| Kobrosly 2014-4 | maternal urine MiBP | CBCL | M+F | ADHD | 6-10 | 153 | 1.1 | 2.6 | 0.1 | 0.9 | 1.3 |
| Kobrosly 2014-43 | maternal urine DEHP | CBCL | M+F | ODD | 6-10 | 153 | 1.0 | 2.9 | 0.1 | 0.9 | 1.1 |
| Kobrosly 2014-7 | maternal urine MiBP | CBCL | M+F | ODD | 6-10 | 153 | 1.2 | 2.5 | 0.2 | 1.0 | 1.4 |

DSM – Diagnostic and Statistical Manual of Mental Disorders; CBCL – Child Behavior Checklist; BASC – Behavior Assessment System for Children; ADHD – Attention Deficient Hyperactivity Disorder; Hyp – hyperactivity; Imp – impulsiveness; Inat – inattention; Extern. Behav. – general externalizing; ODD – Oppositional Defiance Disorder; CD – Conduct Disorder; OR – odds ratio; SE – standard error; V – variance; CI – confidence intervals.

**Table S6.** References and prenatal OC data used in the meta-analyses in this review.

| **Prenatal OC Exposure Data** | | | | | | | | | | |
|---|---|---|---|---|---|---|---|---|---|---|
| **Reference** | **Exposure Period, Matrix and Chemical** | **Mental Health Assessment** | **Outcome** | **Age Range (years)** | ***n*** | **OR** | **SE** | **V** | **Lower 95% CI** | **Upper 95% CI** |
| Capersen 2016-1 | maternal PCB 153 | DSM | ADHD | 2-6 | 1024 | 1.1 | 2.7 | 0.1 | 0.9 | 1.2 |
| Capersen 2016-5 | maternal dioxin like compounds | DSM | ADHD | 2-6 | 1024 | 1.0 | 2.8 | 0.1 | 0.9 | 1.2 |
| Marks 2010-2 | prenatal DAP | CBCL | ADHD | 3.5 | 288 | 1.40 | 1.69 | 0.352 | 0.4 | 3.1 |
| Marks 2010-4 | prenatal DAP | CBCL | ADHD | 5 | 319 | 0.60 | 3.77 | 0.07 | 0.3 | 1.2 |
| Ode 2014-1 | Cord Serum PFOS | DSM | ADHD | 8-12 | 408 | 1.0 | 2.9 | 0.1 | 0.9 | 1.0 |
| Ode 2014-2 | Cord Serum PFOA | DSM | ADHD | 8-12 | 408 | 1.0 | 3.0 | 0.1 | 0.9 | 1.0 |
| Perera 2014-1 | cord Pah adducts | CBCL | ADHD | 9 | 162 | 1.2 | 1.9 | 0.3 | 0.2 | 5.7 |
| Perera 2014-2 | cord Pah adducts | DSM | ADHD | 9 | 162 | 1.7 | 1.4 | 0.5 | 0.5 | 6.2 |
| Ribas-Fito 2007-1 | In utero HCB (.5-.99 ng/ml) | DSM | ADHD | 5 | 405 | 1.6 | 1.5 | 0.4 | 0.5 | 6.5 |
| Ribas-Fito 2007-2 | In utero HCB (1-1.49 ng/ml) | DSM | ADHD | 5 | 405 | 4.1 | 0.7 | 2.1 | 0.9 | -32.6 |
| Ribas-Fito 2007-3 | In utero HCB (1.5+ ng/ml) | DSM | ADHD | 5 | 405 | 6.4 | 0.5 | 3.8 | 1.3 | -15.3 |
| Ribas-Fito 2007-4 | In utero HCB (ng/ml) | DSM | ADHD | 5 | 405 | 1.4 | 2.2 | 0.2 | 1.2 | 5.3 |
| Sagiv 2010-7 | Cord Serum- Sum of 51 PCBS | DSM | ADHD | 8 | 521 | 1.0 | 2.9 | 0.1 | 1.0 | 1.0 |
| Sagiv 2010-8 | Cord Serum- Sum of 4 PCBS | DSM | ADHD | 8 | 521 | 1.1 | 2.8 | 0.1 | 1.0 | 1.1 |
| Sagiv 2010-9 | Cord Serum DDE | DSM | ADHD | 8 | 521 | 1.0 | 2.9 | 0.1 | 1.0 | 1.0 |
| Capersen 2016-4 | maternal PCB 153 | DSM | ADHD Hyp | 2-6 | 1024 | 1.0 | 2.8 | 0.1 | 0.9 | 1.2 |
| Capersen 2016-8 | maternal dioxin like compounds | DSM | ADHD Hyp | 2-6 | 1024 | 1.0 | 2.8 | 0.1 | 0.9 | 1.2 |
| Furlong 2017-4 | maternal Urinary pyrethriods | BASC | ADHD Hyp/Imp | 6-9 | 162 | 0.4 | 5.6 | 0.0 | 0.0 | 14.3 |
| Perera 2014-3 | cord Pah adducts | DSM | ADHD Hyp/Imp | 9 | 162 | 1.0 | 2.2 | 0.2 | 0.3 | 3.2 |
| Sagiv 2010-4 | Cord Serum- Sum of 51 PCBS | DSM | ADHD Hyp/Imp | 8 | 521 | 1.0 | 2.9 | 0.1 | 1.0 | 1.0 |
| Sagiv 2010-5 | Cord Serum- Sum of 4 PCBS | DSM | ADHD Hyp/Imp | 8 | 521 | 1.1 | 2.8 | 0.1 | 1.0 | 1.1 |
| Sagiv 2010-6 | Cord Serum DDE | DSM | ADHD Hyp/Imp | 8 | 521 | 1.0 | 2.9 | 0.1 | 1.0 | 1.0 |
| Capersen 2016-3 | maternal PCB 153 | DSM | ADHD Imp | 2-6 | 1024 | 1.1 | 2.7 | 0.1 | 0.9 | 1.2 |
| Capersen 2016-7 | maternal dioxin like compounds | DSM | ADHD Imp | 2-6 | 1024 | 1.1 | 2.7 | 0.1 | 0.9 | 1.2 |
| Boucher 2012-10 | Cord PCB 153 | DSM | ADHD Inat | 8-14 | 68 | 1.0 | 2.8 | 0.1 | 0.9 | 1.2 |
| Capersen 2016-2 | maternal PCB 153 | DSM | ADHD Inat | 2-6 | 1024 | 1.1 | 2.7 | 0.1 | 0.9 | 1.3 |
| Capersen 2016-6 | maternal dioxin like compounds | DSM | ADHD Inat | 2-6 | 1024 | 1.0 | 2.9 | 0.1 | 0.8 | 1.2 |
| Marks 2010-1 | prenatal DAP | CBCL | ADHD Inat | 3.5 | 289 | 1.60 | 1.73 | 0.336 | 0.8 | 3.5 |
| Marks 2010-3 | prenatal DAP | CBCL | ADHD Inat | 5 | 319 | 1.00 | 2.36 | 0.18 | 0.4 | 2.4 |
| Perera 2014-4 | cord Pah adducts | DSM | ADHD Inat | 9 | 162 | 1.3 | 1.7 | 0.3 | 0.3 | 5.6 |
| Sagiv 2010-1 | Cord Serum- Sum of 51 PCBS | DSM | ADHD Inat | 8 | 521 | 1.0 | 2.9 | 0.1 | 1.0 | 1.0 |
| Sagiv 2010-2 | Cord Serum- Sum of 4 PCBS | DSM | ADHD Inat | 8 | 521 | 1.0 | 2.8 | 0.1 | 1.0 | 1.1 |
| Sagiv 2010-3 | Cord Serum DDE | DSM | ADHD Inat | 8 | 521 | 1.0 | 2.9 | 0.1 | 1.0 | 1.0 |
| Furlong 2017-3 | maternal Urinary pyrethriods | BASC | CD | 6-9 | 162 | 0.1 | 15.1 | 0.0 | 0.0 | 3.0 |
| Furlong 2017-2 | maternal Urinary pyrethriods | BASC | Extern. Behav. | 6-9 | 162 | 0.3 | 7.8 | 0.0 | 0.0 | 5.2 |
| Boucher 2012-11 | Cord PCB 153 | DSM | Extern. Behav. | 8-14 | 268 | 1.0 | 2.8 | 0.1 | 0.9 | 1.1 |
| Furlong 2017-1 | maternal Urinary pyrethriods | BASC | Extern. Behav. | 6-9 | 162 | 0.4 | 4.9 | 0.0 | 0.0 | 9.4 |

DSM – Diagnostic and Statistical Manual of Mental Disorders; CBCL – Child Behavior Checklist; BASC – Behavior Assessment System for Children; ADHD – Attention Deficient Hyperactivity Disorder; Hyp – hyperactivity; Imp – impulsiveness; Inat – inattention; Extern. Behav. – general externalizing; ODD – Oppositional Defiance Disorder; CD – Conduct Disorder; OR – odds ratio; SE – standard error; V – variance; CI – confidence intervals.

**Table S7.** References and prenatal Hg data used in the meta-analyses in this review.

| Prenatal Hg Exposure Data | | | | | | | | | | |
|---|---|---|---|---|---|---|---|---|---|---|
| **Reference** | **Biological Matrix** | **Mental Health Assessment** | **Outcome** | **Age Range (years)** | ***n*** | **OR** | **SE** | **V** | **Lower 95% CI** | **Upper 95% CI** |
| Sagiv 2012-3 | maternal hair hg (< 1ug/g) | DSM | ADHD | 8 | 362 | 0.45 | 1.51 | 0.44 | -2.52 | 3.42 |
| Sagiv 2012-6 | maternal hair hg (1+ug/g) | DSM | ADHD | 8 | 362 | 2.54 | 1.35 | 0.55 | -0.10 | 5.19 |
| Sagiv 2012-2 | maternal hair hg (< 1ug/g) | DSM | ADHD Hyp/Imp | 8 | 363 | 0.55 | 1.32 | 0.57 | -2.04 | 3.14 |
| Sagiv 2012-5 | maternal hair hg (1+ug/g) | DSM | ADHD Hyp/Imp | 8 | 362 | 2.92 | 1.48 | 0.46 | 0.03 | 5.81 |
| Boucher 2012-3 | cord hg | DSM | ADHD Inat | 8-14 | 269 | 1.14 | 2.60 | 0.15 | 1.00 | 1.28 |
| Desrosier 2013-5 | cord hg (low) | CBCL | ADHD Inat | 11 | 131 | 1.16 | 2.47 | 0.16 | 0.90 | 3.28 |
| Desrosier 2013-8 | cord hg (high) | CBCL | ADHD Inat | 11 | 131 | 1.19 | 2.47 | 0.16 | 0.99 | 3.69 |
| Sagiv 2012-1 | maternal hair hg (< 1ug/g) | DSM | ADHD Inat | 8 | 362 | 0.59 | 1.45 | 0.47 | -2.26 | 3.43 |
| Sagiv 2012-4 | maternal hair hg (1+ug/g) | DSM | ADHD Inat | 8 | 362 | 1.93 | 1.33 | 0.56 | -0.68 | 4.55 |
| Boucher 2012-5 | cord hg | DSM | ADHD-HypImp | 8-14 | 181 | 0.95 | 2.38 | 0.18 | 0.30 | 3.00 |
| Boucher 2012-8 | cord hg | DSM | ADHD-HypImp | 8-14 | 178 | 2.92 | 1.04 | 0.93 | 1.07 | 8.04 |
| Boucher 2012-4 | cord hg | DSM | ADHD-Inat | 8-14 | 181 | 2.77 | 1.07 | 0.88 | 1.00 | 7.65 |
| Boucher 2012-7 | cord hg | DSM | ADHD-Inat | 8-14 | 178 | 2.87 | 1.05 | 0.92 | 1.04 | 7.94 |
| Desrosier 2013-4 | cord hg (low) | CBCL | Extern. Behav. | 11 | 131 | 1.28 | 2.52 | 0.16 | 1.27 | 5.07 |
| Desrosier 2013-7 | cord hg (high) | CBCL | Extern. Behav. | 11 | 131 | 1.11 | 2.46 | 0.16 | 0.77 | 2.78 |
| Boucher 2012-2 | cord hg | DSM | Extern. Behav. | 8-14 | 269 | 1.05 | 2.75 | 0.13 | 0.93 | 1.20 |
| Desrosier 2013-3 | cord hg (low) | CBCL | Extern. Behav. | 11 | 131 | 1.31 | 2.55 | 0.15 | 1.38 | 5.64 |
| Desrosier 2013-6 | cord hg (high) | CBCL | Extern. Behav. | 11 | 131 | 1.16 | 2.47 | 0.16 | 0.90 | 3.28 |
| Boucher 2012-1 | cord hg | DSM | ODD CD | 8-14 | 178 | 1.39 | 1.88 | 0.28 | 0.65 | 2.98 |
| Boucher 2012-6 | cord hg | DSM | ODD CD | 8-14 | 181 | 1.19 | 2.12 | 0.22 | 0.56 | 2.56 |

DSM – Diagnostic and Statistical Manual of Mental Disorders; CBCL – Child Behavior Checklist; ADHD – Attention Deficient Hyperactivity Disorder; Hyp – hyperactivity; Imp – impulsiveness; Inat – inattention; Extern. Behav. – general externalizing; ODD – Oppositional Defiance Disorder; CD – Conduct Disorder; OR – odds ratio; SE – standard error; V – variance; CI – confidence intervals.
